# High-density lipoprotein protects normotensive and hypertensive rats against ischemia-reperfusion injury through differential regulation of mTORC1 and mTORC2 signaling

**DOI:** 10.3389/fphar.2024.1398630

**Published:** 2024-11-14

**Authors:** Reham Al-Othman, Aishah Al-Jarallah, Fawzi Babiker

**Affiliations:** ^1^ Department of Biochemistry, Faculty of Medicine, Kuwait University, Kuwait City, Kuwait; ^2^ Department of Physiology, College of Medicine, Kuwait University, Kuwait City, Kuwait

**Keywords:** HDL, mTOR, Akt, ischemia/reperfusion injury, hypertension, apoptosis

## Abstract

**Background:**

High-density lipoprotein (HDL) protects against myocardial ischemia-reperfusion (I/R) injury. Mammalian target of rapamycin complexes 1 and 2 (mTORC1 and mTORC2) play opposing roles in protecting against I/R injury, whereby mTORC1 appears to be detrimental while mTORC2 is protective. However, the role of HDL and mTORC signaling in protecting against I/R in hypertensive rodents is not clearly understood. In this study, we investigated the involvement of mTORC1 and mTORC2 in HDL-mediated protection against myocardial I/R injury in normotensive Wistar Kyoto (WKY) rats and spontaneously hypertensive rats (SHR).

**Methods:**

Hearts from WKY and SHR were subjected to I/R injury using a modified Langendorff system. Hemodynamics data were collected, and infarct size was measured. Rapamycin and JR-AB2-011 were used to test the role of mTORC1 and mTORC2, respectively. MK-2206 was used to test the role of Akt in HDL-mediated cardiac protection. The expression levels and the activation states of mediators of mTORC1 and mTORC2 signaling and myocardial apoptosis were measured by immunoblotting and/or enzyme-linked immunosorbent assay (ELISA).

**Results:**

HDL protected hearts from WKY and SHR against I/R injury as indicated by significant improvements in cardiac hemodynamics and reduction in infarct size. HDL induced greater protection in WKY compared to SHR. HDL treatment attenuated mTORC1 signaling in WKY by reducing the phosphorylation of P70S6K (mTORC1 substrate). In SHR however, HDL attenuated mTORC1 signaling by reducing the levels of phospho-mTORC1, Rag C (mTORC1 activator), and phospho-PRAS40 (mTORC1 inhibitor). HDL increased the phosphorylation of mTORC2 substrate Akt, specifically the Akt2 isoform in SHR and to a greater extent in WKY. HDL-induced protection was abolished in the presence of Akt antagonist and involved attenuation of GSK, caspases 7 and 8 activation, and cytochrome C release.

**Conclusion:**

HDL mediates cardiac protection via attenuation of mTORC1, activation of mTORC2-Akt2, and inhibition of myocardial apoptosis. HDL regulates mTORC1 and mTORC2 signaling via distinct mechanisms in normotensive and hypertensive rats. HDL attenuation of mTORC1 and activation of mTORC2-Akt2 signaling could be a mechanism by which HDL protects against myocardial I/R injury in hypertension.

## Introduction

Hypertension continues to be a key risk factor in the development of cardiovascular diseases (Khan et al., 2020). Hypertension-induced cardiovascular complications involve functional and structural changes in the myocardium and coronary arteries including, but not limited, to increased workload, left ventricular hypertrophy (Yildiz et al., 2020), endothelial dysfunction (Gallo et al., 2021), and enhanced atherosclerotic plaque development (Ruilope and Schmieder, 2008; Li and Chen, 2005) resulting in ischemic heart disease (IHD). Hearts from hypertensive rodents demonstrated a notable resistance to the protection offered by ischemic postconditioning (Wagner et al., 2013; Babiker et al., 2019), erythropoietin (Yano et al., 2011), helium (Oei et al., 2012), and captopril (Penna et al., 2010). We have recently reported that acute and chronic treatment with high-density lipoprotein (HDL) protects hearts from spontaneously hypertensive rats (SHR) against myocardial ischemia-reperfusion (I/R) injury (Al-Jarallah and Babiker, 2022; Al-Jarallah and Babiker, 2024). The cardioprotective effects of HDL in hypertension are however not clearly understood.

Mammalian target of rapamycin complex 1 (mTORC1) and complex 2 (mTORC2) regulate cellular responses to stress conditions including ischemia (Laplante and Sabatini, 2012). mTORC1 inhibition with rapamycin protected against myocardial I/R injury and reduced cardiomyocyte apoptosis (Filippone et al., 2017; Das et al., 2015; Samidurai et al., 2020) suggesting a detrimental role of mTORC1 in mediating myocardial I/R injury. mTORC2 on the other hand, via the activation of protein kinase B (Akt), appears to be cardioprotective (Filippone et al., 2017; Samidurai et al., 2020; Yano et al., 2014). Interestingly, rapamycin-mediated inhibition of mTORC1 reduced blood pressure, albumin secretion and renal inflammatory cell infiltration in Dahl salt-sensitive rats (Kumar et al., 2017). HDL activated phosphatidylinositol-3-kinase (PI3K)/AKt/mTORC signaling and protected against oxidative stress-induced cardiomyocyte apoptosis (Nagao et al., 2017). Nonetheless, the effect of HDL on mTORC1 and mTORC2 in the protection against I/R injury in hypertensive rodents is not clearly understood. We hypothesize that HDL protects against I/R injury by inhibiting mTORC1 and activating mTORC2 in spontaneously hypertensive rats (SHR). We report that mTORC1 and mTORC2 exhibit contrasting functions in mediating myocardial I/R injury. Moreover, we demonstrate that HDL offers protection against I/R injury in normotensive and hypertensive rats to varying degrees. HDL inhibited mTORC1 in normotensive and hypertensive rats via different mechanisms. HDL, however, activated mTORC2 in both genotypes. HDL-mediated protection against I/R injury in WKY and SHR involved attenuation of myocardial apoptosis.

## Materials and methods

### Materials

All materials were purchased from Sigma Aldrich (Germany, Steinheim) unless stated otherwise.

### Animals and instrumentation

Twelve to fourteen-week-old male Wistar Kyoto (WKY) rats and spontaneously hypertensive rats (SHR) were randomized and used in the study (n = 4–9 rats per treatment). The SHR model was chosen because it is a well-established model for studying essential hypertension and hypertension-related physiological and biochemical changes (Al-Jarallah and Babiker, 2024; Dodd et al., 2012). SHR are characterized by elevated blood pressure, autonomic nervous system imbalances cardiovascular and renal complications, making it a valuable tool for understanding the pathophysiology of hypertension and testing potential treatments (Jama et al., 2022; Zhou and Frohlich, 2007). Animals were kept under internationally accepted conditions in the Animal Resource Center, Faculty of Medicine, Kuwait University. All animals were maintained under controlled temperature (21–24 C), 12 h light/dark cycle (light 7 a.m.–7 p.m.) and 50% humidity. The rats were housed in plastic cages (2 rats/cage) with unrestricted access to tap water and food. All procedures were approved by the Health Sciences Research Ethics Committee (ID:3640). Blood pressure was measured using the CODA-4 channel system (Kent Scientific Corporation, United States). Normotensive and hypertensive rats were defined by systolic blood pressure (SBP) cutoff values of ≤120 mmHg and ≥160 mmHg, respectively.

### Experimental procedures and protocols

Heart cannulation and perfusion were performed using a modified Langendorff system as previously described in (Juggi et al., 2011). Briefly, the heart was carefully isolated and immersed in cold (4°C) Krebs-Hensleit (KH) solution. The isolated hearts were perfused retrogradely with a freshly prepared KH buffer mmol/L: NaCl 117.86, KCl 5.59, CaCl_2_.H_2_O 2.4, NaHCO_3_ 20, KH_2_PO_4_ 1.19, MgCl_2_.6H_2_O 1.2, Glucose 12.11. The buffer was gazed with a mixture of O_2_ (95%) and CO_2_ (5%), pH (7.35–7.45) at 37°C. After stabilization (20 min), regional ischemia was induced by occluding the left anterior descending (LAD) coronary artery for 30 min. The success of ischemia induction was evaluated at the onset of ischemia by an immediate drop in the coronary flow. Preload was kept constant at 6 mmHg under basal controlled conditions and perfusion pressure (PP) at 50 mmHg was maintained throughout the experimental procedure. A water-filled latex balloon was placed and secured in the left ventricular (LV) cavity. The balloon was attached to a pressure transducer and a “DC-Bridge amplifier (DC-BA)” with a pressure module (DC-BA type 660, Hugo-Sachs Electronik, Germany) and interfaced to a personal computer for monitoring LV developed pressure (DPmax). LV developed pressure was derived from acquisition of LV end systolic pressure (LVESP) using Max-Min module (Number MMM type 668, Hugo Sachs Elektronik-Harvard Apparatus GmbH, Germany) which converts the output from DC bridge amplifier to DPmax by subtracting LV end diastolic pressure (LVEDP) from the LVESP. All hearts were subjected to ischemia produced by LAD coronary artery occlusion by a snare at ∼0.5 cm below the atrioventricular groove, and a small rigid plastic tube was positioned between the heart and the snare to ensure complete occlusion of the coronary artery.

Hearts were subjected to I/R injury without any treatments (untreated controls, Supplementary Figure S1, group A) or treated with mTORC1 antagonist, rapamycin (100 nM) (Das et al., 2015), mTORC2 specific antagonist JR-AB2-011 (5 µM) (Benavides-Serrato et al., 2017) or Akt antagonist, MK-2206 (5 µM) (Chen et al., 2018) infused at 25 min of ischemia and continued until 10 min of reperfusion (Supplementary Figure S1, group B). Alternatively, hearts were treated with HDL (400 µg) (Lee BioSolutions, United States) (Al-Jarallah and Babiker, 2024) administered 5 min before reperfusion and continued for an additional 10 min (Supplementary Figure S1, group C). In the fourth group, hearts were pretreated with MK-2206 (5 µM) infused 5 min prior to the addition of HDL (400 µg) and continued during the first 10 min of reperfusion (Supplementary Figure S1, group D). At the end of each experiment, hearts were collected, snap-frozen in liquid nitrogen, and stored at −80 °C for further analysis.

### Data collection and processing

Left ventricular function was evaluated by the assessment of LV end diastolic pressure (LVEDP) and DPmax, cardiac contractility was monitored by heart contractility index values (±dp/dt), while coronary-vascular dynamics were evaluated by the coronary flow, measured using an electromagnetic flow probe attached to the inflow of the aortic cannula interfaced to a personal computer. The coronary flow (CF) (mL/min) was continuously monitored using a software developed specifically for this purpose and was manually verified by the timed collection of coronary effluent. The coronary vascular resistance (CVR) and hemodynamics data were determined every 10 s using an online data acquisition program (Isoheart software V 1.524-S, Hugo-Sachs Electronik, Germany).

### Evaluation of infarct size by triphenyltetrazolium chloride staining

Hearts (n = 3) were sliced transversely into 4–6 pieces from apex to base. The slices were incubated in 1% triphenyltetrazolium chloride (TTC) solution in isotonic (pH 7.40) phosphate buffer and fixed in 4% formaldehyde for 24 h. Infarct size was measured blindly using Scion ImageJ (ImageJ, Wayne Rasb and National Institute of Health, United States). The infarcted area of the LV was expressed as a percentage of the total LV area.

### Tissue homogenization and protein extraction

Hearts were homogenized using a polytron homogenizer (Ultra-Turrax: T 25 basic: IKA®-Werk, Germany) in ice cold buffer containing: 0.2x PBS, 0.1% triton-x100, 1x protease inhibitor cocktail, 1x phosphatase inhibitor cocktail, pH (7.40). The hearts were subjected to four homogenization cycles, 30 s each, with 60 s cooling in between. Homogenates were centrifuged at 6,000 rpm for 15 min at 4°C in a benchtop centrifuge (Beckman J2-MI, United States). The supernatant was collected, aliquoted, and stored at −80°C for further analysis. Protein concentration was estimated using a BCA assay kit (Thermo Fisher Scientific, MA, United States) following manufacturer instructions. Absorbance readings were measured at 562 nm (BMG LabTech ClarioStar, Germany).

### Immunoblotting

Protein expression was detected using sodium dodecyl sulfate-polyacrylamide gel electrophoresis (SDS-PAGE) followed by immunoblotting against target proteins. Samples (50 µg protein) mixed with the loading buffer were boiled for 5 min and loaded into 4%–20% gradient Tris-glycine polyacrylamide gels (BioRad, United States). Proteins were then transferred to polyvinylidene difluoride (PVDF) membranes. Membranes were blocked with 5% nonfat dairy milk (NFDM) or 5% bovine serum albumin (BSA) in Tris-buffered saline, 0.1% Tween (TBS-T) for 1 h at room temperature. Membranes were blotted with primary antibodies against phospho-mTOR (Ser2448), total mTOR, phosphorylated-40 kDa proline-rich AKT substrate (PRAS40) (Thr246), total PRAS40, phosphorylated-ribosomal protein S6 kinase beta-1 (P70S6K) (Thr 389), total P70S6K, phosphorylated-eukaryotic translation initiation factor 4E (eIF4E)-binding protein (4E-BP1) (Thr37/46) and total 4E-BP1, Ras-related GTP-binding protein C (RagC), phospho-Akt (Ser473), total Akt, phosphorylated-Akt1 (Ser473), total Akt1, phosphorylated-Akt2 (Ser474), total Akt2, phosphorylated-glycogen synthase kinase (GSK)-3β (Ser9), total GSK-3β, caspase-7, GAPDH (Cell Signaling, MA, United States) or caspase-8 (Santacruz, United States), overnight at 4°C, followed by horseradish peroxidase (HRP)-conjugated donkey anti-rabbit or donkey anti-mouse antibodies (Jackson ImmunoResearch, United States). Bands were developed using enhanced chemiluminescence (ECL) reagent (Bio-Rad, United States) and detected using Bio-Rad Chemidoc (Bio-Rad chemi-Doc™ MP Imaging System, United States). Bands were quantified using Image Lab software (Bio-Rad, United States).

### Measurements of cytochrome C release

Cytochrome c release was measured in heart homogenates using a commercially available kit from Abcam (ab210575) following the manufacturer’s protocol. Briefly, heart homogenates were diluted (200 x), added to wells precoated with cytochrome c antibody cocktail, and incubated for 1 h at room temperature on a plate shaker. The reaction was then developed by the addition of a substrate solution for 10 min followed by the addition of the stop solution. Cytochrome c levels were determined by measuring the absorbance at 450 nm (BMG LabTech ClarioStar, Germany) and plotting the obtained values against the cytochrome c standard provided with the kit.

### Statistical analysis

Data are presented as means ± *standard error* of the *mean (SEM)*. A two-way analysis of variance (ANOVA) followed by *post hoc* analysis using Bonferroni test was used to test the difference between the means of multiple groups (GraphPad Prism 10.0.2). The two-tailed unpaired student t-test was used to test the significance between two groups that followed a normal distribution while the Mann–Whitney *U* test was used to compare two groups that failed to follow the normal distribution. Differences were considered statically significant at P < 0.05.

## Results

### mTORC1 and mTORC2 play opposing roles in mediating myocardial I/R injury in normotensive and hypertensive rats

Hearts from SHR demonstrated signs of cardiac enlargement (Table 1). SHR had significantly higher (P < 0.01) SBP and diastolic blood pressure (DBP) relative to WKY. In addition, SHR exhibited significantly (P < 0.01) higher heart rate, blood flow, and volume relative to WKY (Table 1).

**TABLE 1:** Heart and body weights of WKY and SHR, and CODA 4-channel high throughput non-invasive blood pressure measurement data.

	WKY	SHR
Body weight (g)	324.7 ± 6.090	279.5 ± 3.832****
Heart Weight (g)	1.667 ± 0.02619	1.551 ± 0.02530**
Heart weight/Body weight	5.23E-03 ± 1.10E-04	5.59E-03 ± 9.18E-05*
Systolic Blood Pressure (mmHg)	113.8 ± 1.277	180.0 ± 1.368****
Diastolic Blood Pressure (mmHg)	74.05 ± 1.013	122.2 ± 1.663****
Mean	86.95 ± 1.079	141.1 ± 1.538****
Rate (Plus/min)	167.8 ± 4.871	291.3 ± 4.572****
Flow (µL/min)	5.787 ± 0.4262	9.023 ± 0.3133****
Volume (µL)	32.36 ± 2.472	62.24 ± 2.058****

^*^P vs. WKY (P < 0.05).

^**^P vs. WKY (P < 0.01).

^****^P vs. WKY (P < 0.0001).

**FIGURE 1:**
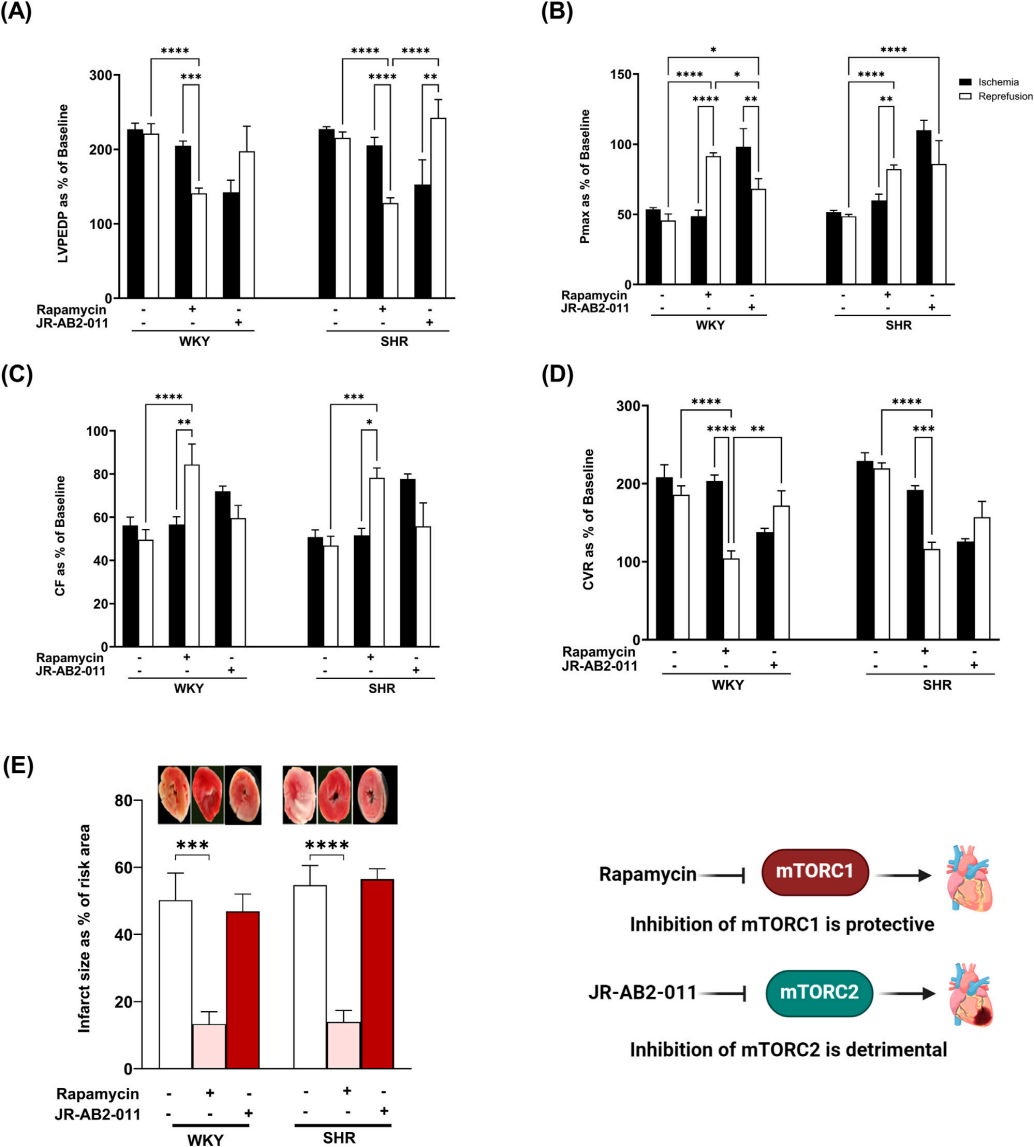
The role of mTORC1 and mTORC2 in mediating I/R injury in WKY and SHR. Post-ischemic recovery parameters of cardiac functions including left ventricular functions (LVPEDP **(A)**, Pmax **(B)**) and coronary hemodynamic (CF **(C)**, CVR **(D)**). Data were computed at 30 min of reperfusion and presented as means ± SEM of n = 4–9 rats per group. Infarct size determination by TTC staining on (n = 3) rats per group **(E)**. *P < 0.05, **P< 0.01, ***P< 0.001, ****P< 0.0001, LVEDP, left ventricular end diastolic pressure; Pmax, maximum developed pressure; CF, coronary flow; CVR, coronary vascular resistance.

Inhibition of mTORC1 with rapamycin protected rodents against myocardial I/R injury (Filippone et al., 2017). Hearts from hypertensive rodents were shown to be resistant to protection induced by pharmacological agents proven, otherwise, to be protective in normotensive rodents (Babiker et al., 2019). The involvement of mTORC1 in mediating I/R injury in SHR has not been previously investigated, we therefore tested if mTORC1 inhibition with rapamycin can protect hypertensive rats from myocardial I/R injury. Rapamycin treatment significantly (P < 0.05) improved LVEDP and Pmax (Figures 1A,B) compared to the respective ischemic period and untreated controls in WKY and SHR. On the other hand, infusion of JR-AB2-011 significantly (P < 0.05) increased LVEDP in SHR and decreased Pmax in WKY and SHR. Moreover, rapamycin significantly (P < 0.05) increased the contractility index ± dP/dt (Table 2) and CF and decreased CVR compared to the respective ischemic period and untreated controls (Figures 1C,D) in WKY and SHR. In addition, rapamycin treatment reduced infarct size in normotensive and hypertensive rats (Figure 1E). This data suggests that mTORC1 plays a detrimental role in mediating I/R injury and inhibition of mTORC1 is protective in normotensive and hypertensive rats. To test the role of mTORC2 we used mTORC2 specific antagonist JR-AB2-011 (Benavides-Serrato et al., 2017; Guenzle et al., 2020). Administration of JR-AB2-011 (5 µM) did not improve cardiac functions in WKY and SHR evident by the persistent deterioration in LV function (Figures 1A,B), cardiac contractility, (Table 2), and coronary vascular dynamics, (Figures 1C,D), compared to the respective ischemic period and untreated control, neither it reduced the infarct size (Figure 1E) suggesting that mTORC2 plays a protective role in WKY and SHR. Collectively this data suggest that mTORC1 and mTORC2 play opposing roles in mediating myocardial I/R injury in normotensive and hypertensive rats.

**TABLE 2:** Cardiac contractility in normotensive and hypertensive rats subjected to different treatments.

	WKY				SHR			
Treatment	+dp/dt		-dp/dt		+dp/dt		-dp/dt	
	Ischemia	Reperfusion	Ischemia	Reperfusion	Ischemia	Reperfusion	Ischemia	Reperfusion
Control	51.5 ± 2.3	53.5 ± 6.5	52.3 ± 2.95	49.7 ± 4.3	50.5 ± 1.7	46.2 ± 2.6	51.6 ± 1.8	49.3 ± 1.4
Rapamycin	52.0 ± 6.1	90.7 ± 7.4^$$$$####^	50.9 ± 5.1	86.7 ± 7.7^$$$####^	67.9 ± 3.7	95.9 ± 4.1^$####^	64.8 ± 7.6	91.3 ± 9.1^$####^
JR-AB-011	101.2 ± 6.1	94.2 ± 12.7^###^	96.8 ± 8.6	79.6 ± 9.0^#^	106.3 ± 10.4	86.5 ± 13.1^##^	104.5 ± 8.7	89.0 ± 9.2^##^
HDL	60.9 ± 6.4	95.2 ± 5.3^$$$$####^	55.4 ± 4.1	96.8 ± 8.2^$$$$####^	48.3 ± 2.2	71.4 ± 3.9^$##*^	48.7 ± 2.0	68.5 ± 1.1^$##***^
MK-0226	65.4 ± 4.1	42.1 ± 6.6	53.0 ± 3.9	39.5 ± 5.7	57.3 ± 3.9	51.7 ± 4.1	41.0 ± 3.1	46.6 ± 1.7
MK-0226+HDL	54.3 ± 4.8	51.2 ± 7.9	42.1 ± 4.8	32.6 ± 4.6^••••^	45.8 ± 3.3	50.2 ± 4.8	42.7 ± 7.3	36.5 ± 7.3^•••^

$p vs. Ischemia (P < 0.05).

^$$$^p vs. Ischemia (P < 0.001).

^$$$$^p vs. Ischemia (P < 0.0001).

#P vs. Control (P < 0.05).

^##^P vs. Control (P < 0.01).

^###^P vs. Control (P < 0.001).

^####^P vs. Control (P < 0.0001).

*P vs. the same treatment in WKY (P < 0.05).

^***^P vs. the same treatment in WKY (P < 0.001).

^•••^P vs. HDL, in the same genotype (P < 0.001).

^••••^P vs. HDL, in the same genotype (P < 0.0001).

### HDL protects against myocardial I/R injury by selectively inhibiting mTORC1 and activating mTORC2 signaling

**FIGURE 2:**
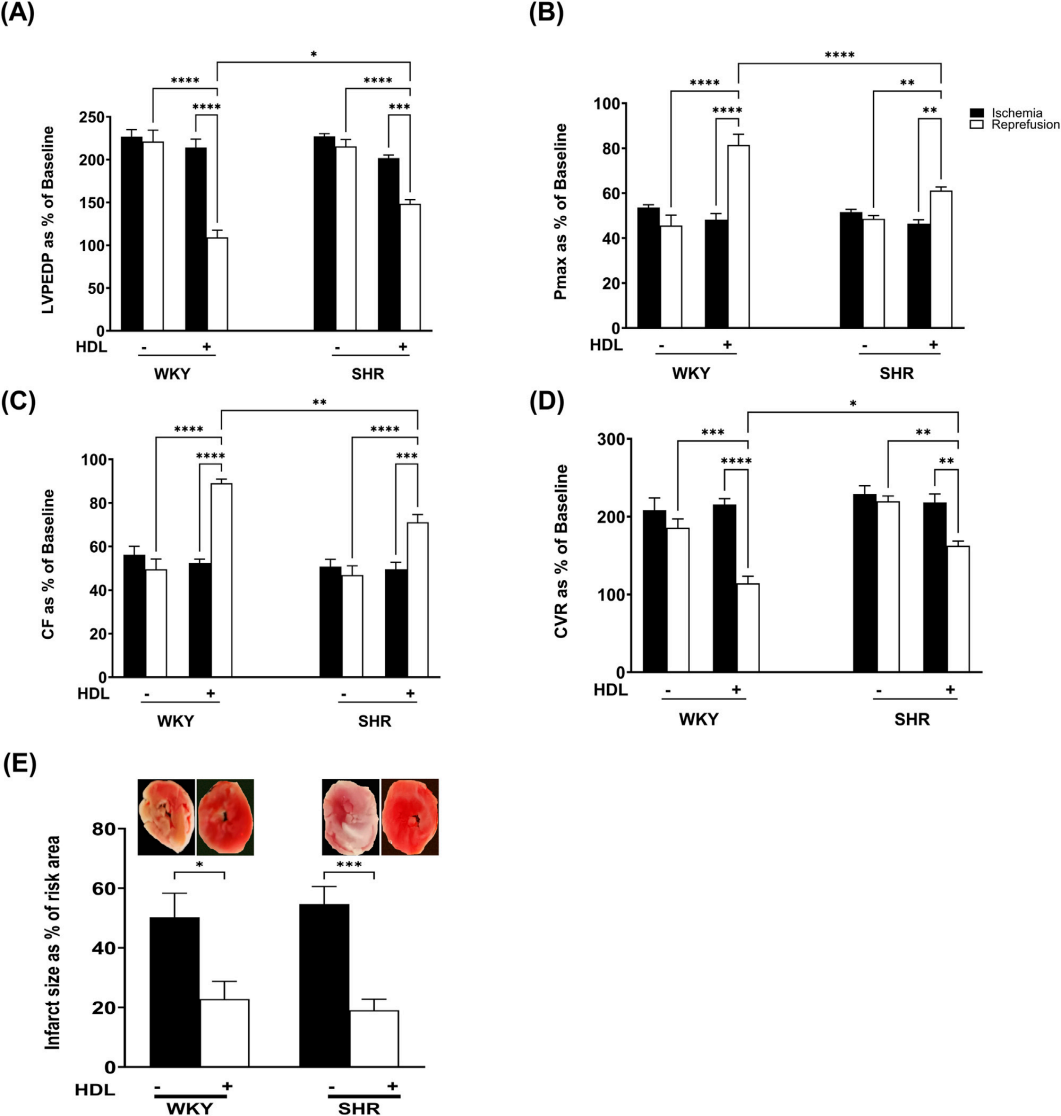
HDL protects WKY and SHR against myocardial I/R injury. Post-ischemic recovery parameters of cardiac functions including left ventricular functions (LVPEDP **(A)**, Pmax **(B)**) and coronary hemodynamic (CF **(C)**, CVR **(D)**). Data were computed at 30 min of reperfusion and presented as means ± SEM of n = 9 rats per group. Infarct size determination by TTC staining (n = 3) rats per group **(E)**. *P < 0.05, **P< 0.01, ***P< 0.001, ****P< 0.0001, LVEDP, left ventricular end diastolic pressure; Pmax, maximum developed pressure; CF, coronary flow; CVR, coronary vascular resistance.

We tested the effect of HDL on I/R-induced myocardial injury in hearts isolated from WKY and SHR. HDL administration, 5 minutes before reperfusion, protected hearts from WKY and SHR from myocardial I/R injury (Figure 2). This was evident by the significant (P < 0.05) improvements in LV functions (LVEDP, Pmax) (Figures 2A,B) cardiac contractility (±dp/dt), (Table 2), and coronary hemodynamics (CF, CVR) (Figures 2C,D) relative to ischemia and relative to untreated controls. Interestingly, HDL induced significantly (P < 0.05) greater protection in WKY relative to SHR, possibly suggesting differences in HDL-mediated signaling between WKY and SHR. Consistent with the protection observed in the physiological parameters we tested, HDL reduced the infarct size in both genotypes (Figure 2E).

**FIGURE 3:**
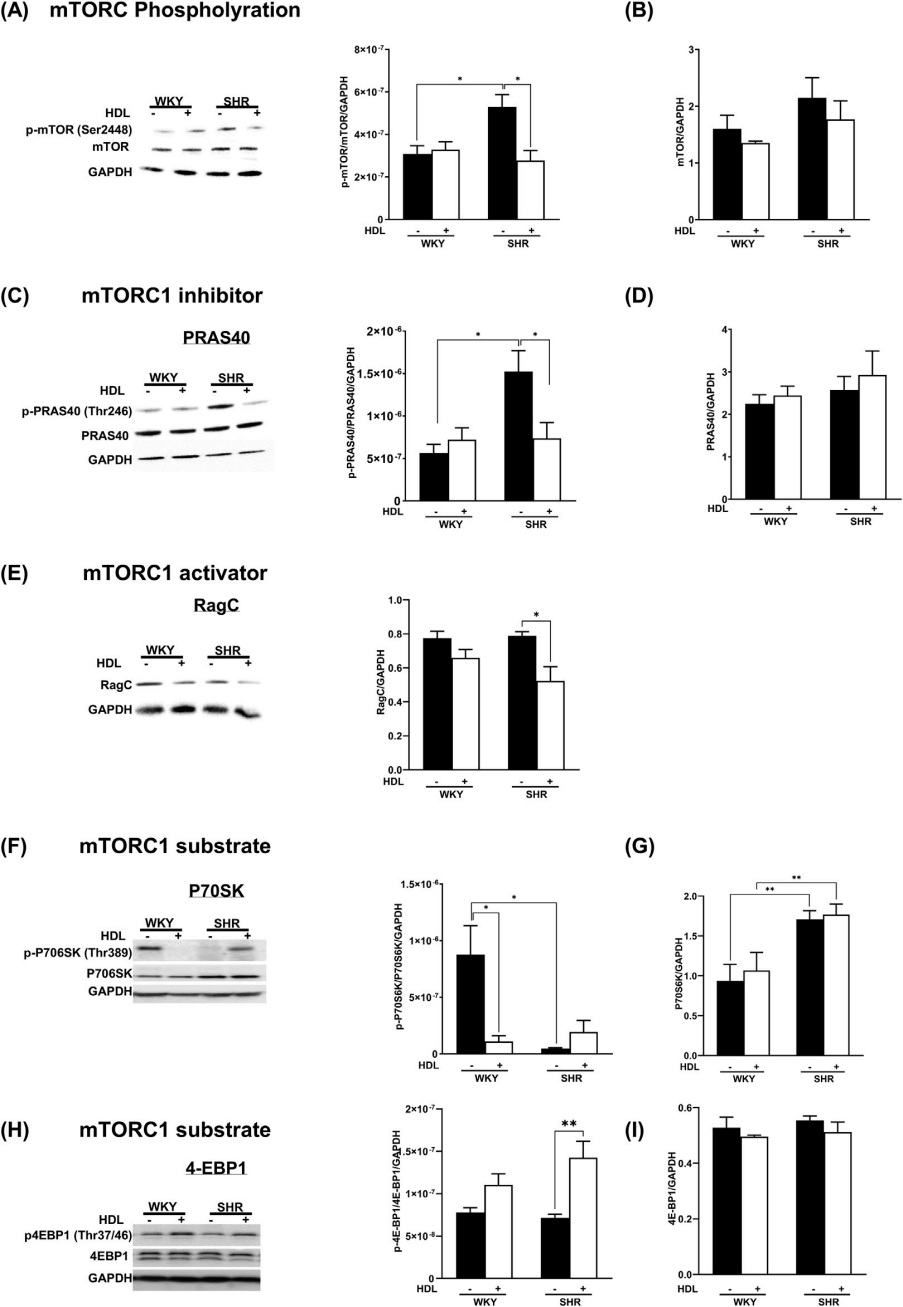
HDL inhibits mTORC1 signaling in WKY and SHR. Hearts from WKY and SHR subjected to I/R injury in the presence or absence of HDL were immunoblotted against mediators of mTORC1 signaling cascade: phospho-mTOR **(A)**, total mTORC **(B)**, phospho-PRAS40 **(C)**, total PRAS40 **(D)**, Rag C **(E)**, phospho-P70S6K **(F)**, total P70S6K **(G)**, phospho-4EBP1 **(H)**, total 4EBP1 **(I)**, and GAPDH as a loading control. Data are means ± SEM. *P < 0.05, **P< 0.01, ***P< 0.001, ****P< 0.0001, n = 3-6.

To test the effects of HDL on the mTORC1 signaling pathway we measured the activation state of mTORC1, mTORC1 substrates, P70S6K, 4E-BP1, and mTORC1 inhibitor, PRAS40. In addition, we examined the expression levels of mTORC1 activator, Rag C in heart homogenates from WKY and SHR treated with or without HDL. SHR demonstrated significantly (P < 0.05) higher basal levels of mTOR phosphorylation at Ser2448, a site predominantly phosphorylated in mTORC1 (Copp et al., 2009) (Figure 3A). HDL treatment significantly (P < 0.05) reduced Ser2448 phosphorylation in SHR but did not have any significant effects in WKY (Figure 3A). This data suggests enhanced basal activation of mTORC1 in SHR that is significantly reduced by HDL treatment.

The binding of PRAS40 to mTORC1 results in complex inhibition (Oshiro et al., 2007). The phosphorylation of PRAS40 by Akt, however, results in its dissociation from the complex and alleviation of inhibition (Sancak et al., 2007; Wang et al., 2007). SHR expressed significantly (P < 0.05) higher basal levels of phospho-PRAS40 compared to WKY (Figure 3C), indicating the presence of increased levels of active mTORC1-PRAS40-free in SHR. HDL treatment significantly (P < 0.05) reduced PRAS40 phosphorylation in SHR, however, it did not change the phosphorylation state of PRAS40 in WKY (Figure 3C). Moreover, HDL treatment did not affect total PRAS40 expression in WKY and SHR (Figure 3D). Similar levels of total-PRAS40 were detected in hearts from normotensive and hypertensive rats. Furthermore, we examined the protein levels of mTORC1 activator, Rag C (Figure 3E). Basal protein levels of Rag C were not significantly (P < 0.05) different between WKY, and SHR. HDL did not affect Rag C protein levels in WKY, yet it significantly (P < 0.05) reduced Rag C expression in SHR (Figure 3E). Finally, we tested the effect of HDL on the activation state of the mTORC1 substrate, P70S6K. (Figure 3F). WKY expressed significantly (P < 0.05) higher basal levels of phospho-P70S6K compared to SHR (Figure 3F). HDL treatment significantly (P < 0.05) reduced P70S6K phosphorylation in WKY. Total P70S6K protein levels were significantly (P < 0.05) higher in SHR than in WKY (Figure 3G). HDL treatment, however, did not affect total P70S6K levels in WKY or SHR (Figure 3G). The HDL treatment significantly (P < 0.05) increased levels of phospho-4E-BP1 in SHR but not in WKY (Figure 3H). To summarize, immunoblotting experiments revealed that HDL has an inhibitory effect on mTORC1 signaling in WKY and SHR. The mechanism of HDL-mediated inhibition of mTORC1 appears to be different between normotensive and hypertensive rats. In WKY, HDL reduced the levels of phospho-P70S6K. In SHR however, HDL decreased PRAS40 phosphorylation and Rag C protein levels (Figures 3A–G). Together this suggests that HDL-mediated inhibition of mTORC1 could be one mechanism by which HDL protects against I/R injury in WKY and SHR. Nonetheless, HDL appears to differentially regulate mediators of mTORC1 signaling in WKY and SHR.

**FIGURE 4:**
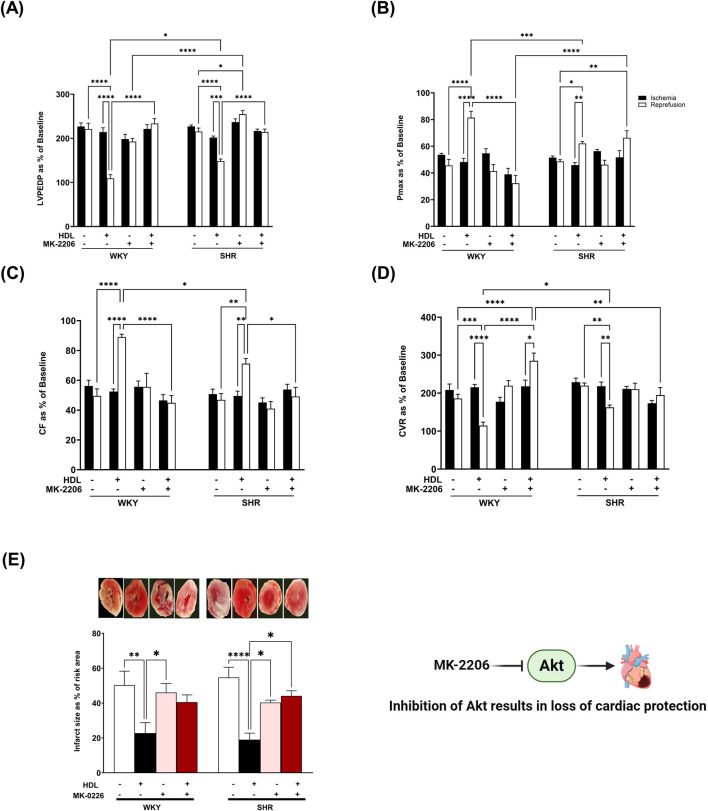
HDL-mediated cardiac protection requires Akt. Post-ischemic recovery parameters of cardiac functions including left ventricular functions (LVPEDP **(A)**, Pmax **(B)**) and coronary hemodynamic (CF **(C)**, CVR **(D)**). Data were computed at 30 min of reperfusion and presented as means ± SEM of n = 9 rats per group. Infarct size determination by TTC staining (n = 3) rats per group **(E)**. *P < 0.05, **P< 0.01, ***P< 0.001, ****P< 0.0001, LVEDP, left ventricular end diastolic pressure; Pmax, maximum developed pressure; CF, coronary flow; CVR, coronary vascular resistance.

Protein kinase B (Akt) is a downstream target of mTORC2 (Oh and Jacinto, 2011). To test the effect of HDL on mTORC2 signaling we used Akt specific antagonist MK-2206 (Chen et al., 2018; Akhtar and Jabeen, 2018) and examined the phosphorylation state of specific Akt isoforms in response to HDL treatment. MK-2206 infusion did not protect the heart against I/R injury in WKY and SHR as indicated by impaired LV function (Figures 4A,B), cardiac contractility (Table 2), and coronary vascular dynamics (Figures 4C,D) and the lack of change in infarct size (Figure 4E) relative to the control. MK-2206 treatment however, abolished the protective effects of HDL in WKY and SHR (Figure 4). This was consistent with the infarct size data whereby HDL did not reduce the infarct size in MK-2206 treated WKY or SHR (Figure 4E).

**FIGURE 5:**
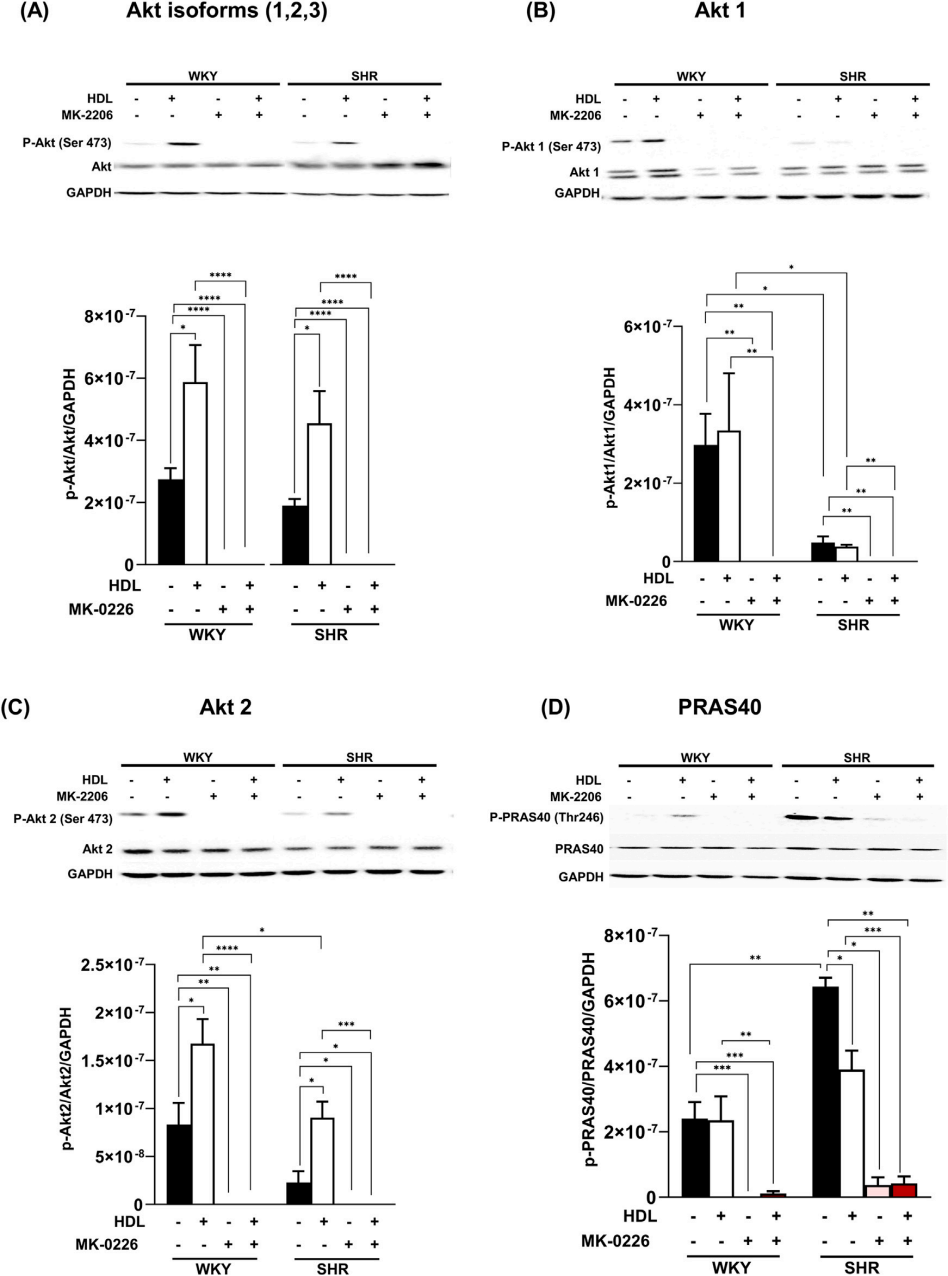
HDL induces the activation of Akt2 isoform. Heart homogenates from WKY and SHR subjected to I/R injury, treated with MK-2206 in the presence or absence of HDL, were subjected to immunoblotting against phospho-Akt, phospho-Akt1 or phospho-Akt2, total Akt, total Akt1, or total Akt2 **(A–C)** or phosphorylated and total PRAS40 **(D)** and GAPDH as a loading control. Data are means ± SEM, *P < 0.05, **P< 0.01, ***P< 0.001, ****P< 0.0001, n = 3–6.

Three Akt isoforms have been identified (Kumar and Madison, 2005; Yu et al., 2015), of which Akt1 and Akt2 are predominantly expressed in the myocardium (Abeyrathna and Su, 2015; Muslin, 2011). We examined the effect of HDL on the phosphorylation of these isoforms. HDL treatment significantly increased (P < 0.05) total Akt phosphorylation at Ser473 in WKY and SHR which was completely abolished in the presence of Akt antagonist (Figure 5A). Interestingly, HDL treatment did not increase Akt1 phosphorylation (Figure 5B), yet it significantly (P < 0.05) increased Akt2 phosphorylation in WKY and SHR (Figure 5C). Furthermore, HDL induced significantly (P < 0.05) greater activation of Akt2 in WKY relative to SHR. Treatment with Akt inhibitor abolished total, non-isoform specific, Akt (Figure 5A), Akt1 (Figure 5B), and Akt2 (Figure 5C) phosphorylation in the presence or absence of HDL. Moreover, Akt inhibition reduced the phosphorylation of Akt substrate, PRAS40 (Figure 5D) in WKY and SHR by 100% and 94.3%, respectively. Akt inhibition reduced PRAS40 phosphorylation by 95.4% and 93.3% in HDL-treated WKY and SHR, respectively (Figure 5D). Together this data suggests that Akt is an essential signaling mediator downstream of HDL that is involved in HDL-mediated cardiac protection. HDL specifically induced Akt2 activation in a magnitude that was proportional to the level of HDL-mediated cardiac protection in normotensive and hypertensive rats. Enhanced HDL-induced activation of Akt2, and enhanced HDL mediated cardiac protection were observed in WKY relative to SHR. Moreover, our data suggest that PRAS40 is a downstream target of Akt in WKY and SHR. HDL appears to phosphorylate PRAS40 through Akt dependent (major) and independent (minor) mechanisms in SHR.

### HDL protects against I/R injury by inhibiting myocardial apoptosis

**FIGURE 6:**
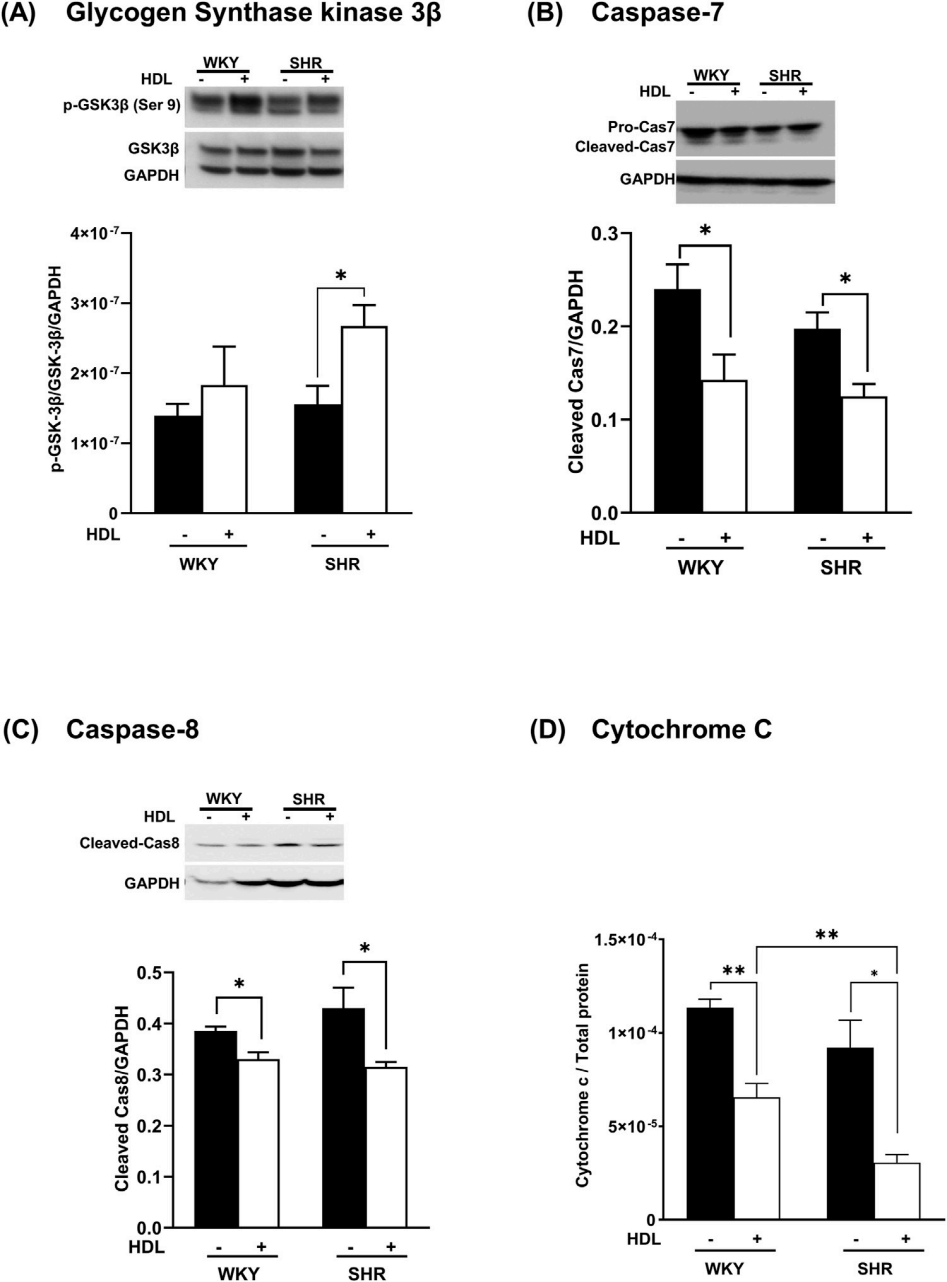
HDL reduces markers of myocardial apoptosis. Heart homogenates from WKY and SHR, subjected to I/R injury in the presence or absence of HDL, were subjected to immunoblotting against, phospho-GSK-3β (Ser9), and total GSK-3β **(A)**, procaspase −7, cleaved caspase 7 **(B)**, cleaved caspase 8 **(C)** and GAPDH as a loading control, and were subjected to ELISA against cytochrome c **(D)**. Data are means ± SEM, *P < 0.05, **P< 0.01, ***P< 0.001, ****P< 0.0001, n = 3-6.

The phosphorylation of Akt inhibits GSK-3β and attenuates myocardial apoptosis (Liu et al., 2020; Huang et al., 2016; Murphy and Steenbergen, 2005). We demonstrate that HDL treatment significantly enhanced GSK-3β phosphorylation (Figure 6A) in SHR with a trend towards an increase in WKY. Furthermore, HDL administration reduced the levels of caspase 7 (Figure 6B) and caspase 8 (Figure 6C) and significantly reduced cytochrome c release (Figure 6D) in heart homogenates from normotensive and hypertensive rats. Together these data suggest that HDL attenuates pathways involved in cardiomyocyte apoptosis. HDL attenuation of cardiomyocyte apoptosis could be one mechanism by which HDL protects against myocardial I/R injury in normotensive and hypertensive rats.

## Discussion

**FIGURE 7:**
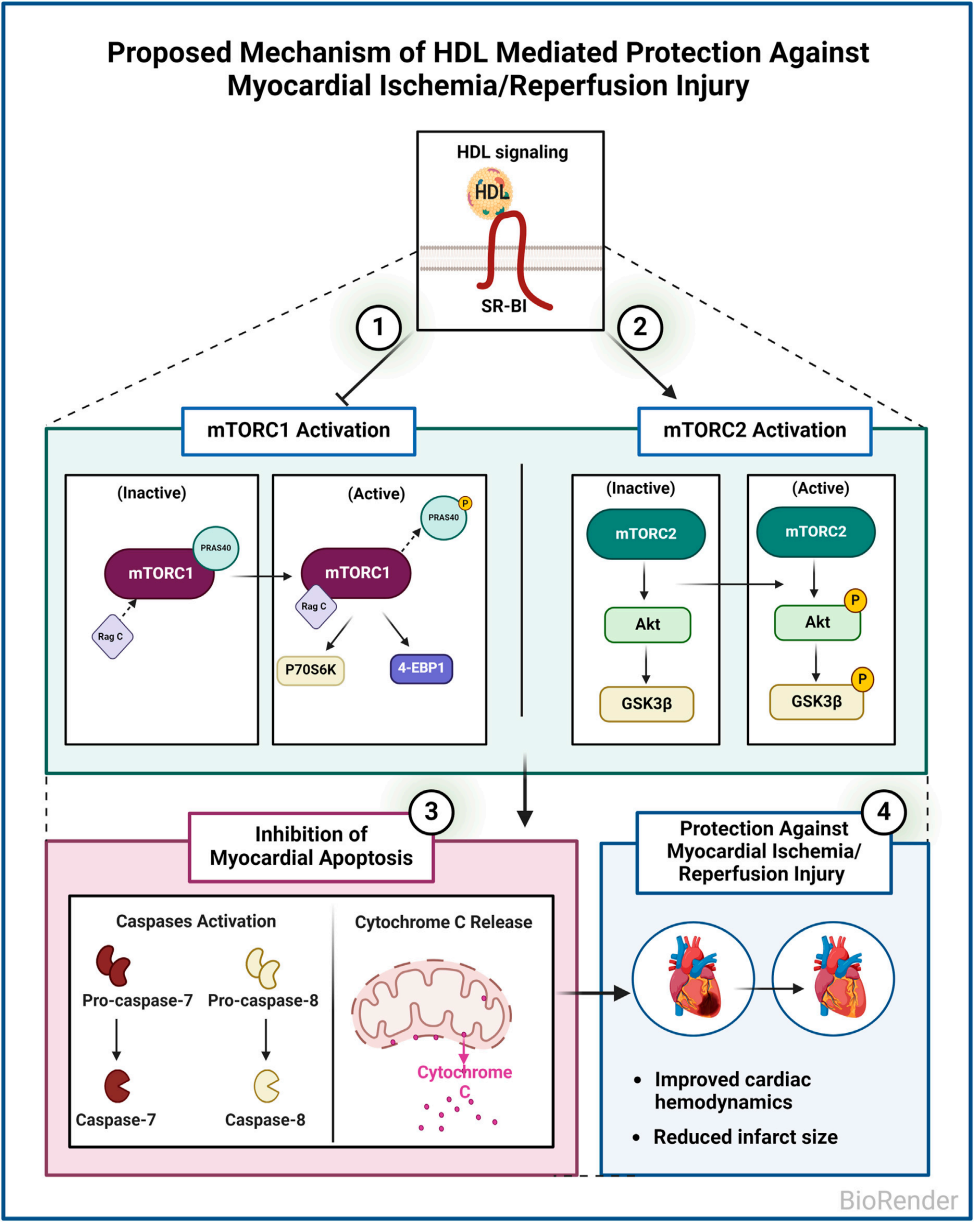
Proposed Mechanism of HDL mediated protection against myocardial I/R injury in WKY and SHR. HDL inhibits mTORC1, activates mTORC2, and inhibits myocardial apoptosis in WKY and SHR. HDL-mediated inhibition of myocardial apoptosis could be one mechanism by which HDL protects against I/R injury in normotensive and hypertensive rats.

In this study, we investigated the involvement of mTORC1 and mTORC2 signaling in HDL-mediated cardiac protection in normotensive and hypertensive rats. We demonstrate that mTORC1 and mTORC2 play opposing roles in mediating myocardial I/R injury. Furthermore, we show that HDL protects against I/R injury in normotensive and hypertensive rats to different extents. HDL inhibits mTORC1 and activates mTORC2 signaling and attenuates myocardial apoptosis following I/R injury (Figure 7).

Mammalian target of rapamycin (mTOR) is present in two complexes, the rapamycin-sensitive mTORC1 and the rapamycin-insensitive mTORC2 (Loewith et al., 2002). mTORC1 regulates protein synthesis, cellular growth, proliferation, ribosomal and mitochondrial biogenesis, autophagy, and metabolism (Johnson et al., 2013; Wullschleger et al., 2006). mTORC1 form a complex with mammalian lethal with SEC13 protein 8 (mLST8), DEP domain-containing mTOR-interacting protein (deptor), PRAS40, tti1/tel2 and regulatory-associated protein of regulatory-associated protein of mammalian target of rapamycin (raptor) (Sciarretta et al., 2014). PRAS40 inhibits complex activity, however, upon phosphorylation it dissociates resulting in the alleviation of the complex (Oshiro et al., 2007; Nascimento and Ouwens, 2009). On the other hand, mTORC1 is activated by Rag GTPases. Rag GTPases form heterodimers whereby Rag A or Rag B interact with Rag C or Rag D (Kim et al., 2008). Active mTORC1 phosphorylates and activates p70S6K which then phosphorylates ribosomal protein S6 and inhibits the binding of 4E-BP1 to eIF4E (Choo et al., 2008; Pullen and Thomas, 1997). mTORC2 however, is composed of the following subunits: SEC13 protein 8, deptor, sin 1, tti1/tel2 and rapamycin-insensitive companion of mTOR (rictor). mTORC2 activates Akt (Sarbassov et al., 2005) and inhibits apoptosis (Filippone et al., 2017). Our data suggests that mTORC1 plays a detrimental role while mTORC2 plays a protective role in mediating myocardial I/R injury in WKY and SHR (Figure 1; Table 2). In addition, hearts from normotensive and hypertensive rats expressed significantly different basal levels of mTORC1 signaling mediators. SHR expressed significantly higher basal levels of phosphorylated-mTOR, phosphorylated-PRAS40, and total-P70S6K, while WKY expressed significantly higher basal levels of phosphorylated-P70S6K (Figure 3). WKY and SHR also expressed significantly different levels of mTORC2 substrates, Akt1 and Akt2. These differences in the basal expression level or activation states of mTORC1 and mTORC2 signaling mediators may suggest differences in the function and/or contribution of these cascades between WKY and SHR which awaits further investigations.

We demonstrate that short-term treatment of HDL protects against myocardial I/R injury in normotensive and hypertensive rats as indicated by improvements in cardiac functions, coronary hemodynamics, and reduction in infarct size (Figure 2; Table 2). Consistent with our previously reported data (Al-Jarallah and Babiker, 2022), HDL was more protective in WKY than it was in SHR (Figure 2; Table 2). The finding that HDL is protective when administered at reperfusion suggests that HDL may represent a promising target for the treatment of ischemic heart disease in normotensive and hypertensive patients. Our findings align with previous reports demonstrating the cardioprotective effects of HDL against ischemic injury (Calabresi et al., 2003; Frias et al., 2013; Gomaraschi et al., 2016). However, the protective mechanisms of HDL against myocardial I/R injury appear to be complex and multifaceted (Durham et al., 2018; Pedretti et al., 2019; White et al., 2016).

We report that HDL inhibited mTORC1 signaling in WKY and SHR (Figure 3). Nonetheless, the mechanism of HDL-mediated inhibition of mTORC1 appears to be different between normotensive and hypertensive rats (Figure 3). In WKY, HDL significantly reduced the level of phospho-P70S6K but did not affect the activation state of mTORC or PRAS40, neither it affected the expression of Rag C. HDL-mediated reduction in P70S6K phosphorylation implicates a reduction in mTORC1 activity in response to HDL, despite of the lack of change in the phosphorylation state of mTORC at Ser2448. HDL treatment in SHR however, reduced the levels of phospho-mTORC, phospho-PRAS40 (inactive inhibitor of mTORC1), and Rag C (mTORC1 activator). To our surprise, HDL did not affect the levels of phospho-P70S6K in hearts from hypertensive rats, possibly suggesting the involvement of other substrates downstream of mTORC1 in response to HDL treatment in these rats. In addition to P70S6K, mTORC1 directly phosphorylates 4E-BP1 (Kazi et al., 2011). HDL treatment increased 4E-BP1 phosphorylation (Figure 3H). Nonetheless, this could be due to mTORC1-indenpendent signaling (Qin et al., 2016). To conclude, in WKY P70S6K appears to be a key downstream substrate of mTORC1, and HDL inhibited mTORC1 by reducing the levels of phosphorylated P70S6K. In SHR however, P70S6K activation seemed to be less significant, despite the increase in basal total levels of P70S6K. Moreover, the mechanism of HDL-mediated inhibition of mTORC1 in SHR involved modulation of mTORC1 activator (Rag C) and inhibitor (PRAS40) suggesting the existence of different mechanisms by which HDL inhibited mTORC1 in WKY and SHR.

In addition, HDL-mediated cardiac protection involved the activation of mTORC2 signaling as indicated by enhanced phosphorylation of mTORC2 substrate, Akt (Oh and Jacinto, 2011; Jacinto et al., 2006), in normotensive and hypertensive rats (Figure 5). This is consistent with the previously reported effect of reconstituted HDL on the activation of mTORC2 in angiogenic cells (Guo et al., 2011). Furthermore, our data is consistent with HDL-mediated activation of Akt in protecting against oxidative damage induced cardiomyocyte necrosis (Durham et al., 2018). Together this suggests that HDL-mediated inhibition of mTORC1 and activation of mTORC2 signaling could be one mechanism by which HDL protects against I/R injury in WKY and SHR.

We further examined the requirement of Akt in HDL-mediated cardiac protection using Akt antagonist, MK-2206. Treatment with MK-2206 abolished HDL-induced improvements in cardiac functions, coronary vascular dynamics (Figures 4A–D; Table 2), and reduction in infarct size (Figure 4E) in WKY and SHR suggesting the requirement of Akt in HDL-induced cardiac protection.

Three Akt isoforms exist of which, Akt1 and Akt2 are the predominant isoforms expressed in the myocardium (Matsui and Rosenzweig, 2005). The lack of Akt1 on an apolipoprotein E knockout background induced features of plaque vulnerability and cardiac dysfunction (Fernandez-Hernando et al., 2009). Moreover, Akt1 played an essential role in mediating physiological cardiac growth and attenuated pathological cardiac hypertrophy (DeBosch et al., 2006a). Akt2 however, was dispensable in maintaining cardiac phenotype (Cho et al., 2001). Nonetheless, Akt2 regulated cardiac glucose metabolism and survival (DeBosch et al., 2006b).

A considerable amount of interaction between mTORC1 and mTORC2 has been reported. For instance, mTORC1-induced activation of P70S6K suppresses mTORC2 (Fu and Hall, 2020; Harrington et al., 2004). In addition, Akt mediates a positive activation loop between mTORC1 and mTORC2 whereby mTORC2 activates Akt (Abeyrathna and Su, 2015), which then alleviates mTORC1 inhibition by phosphorylating PRAS40 (Wang et al., 2007). Treatment with Akt antagonist, MK-2206, abolished the phosphorylation of total, non-isoform specific, Akt, Akt1, and Akt2 and Akt target, PRAS40 (Figure 5). This is consistent with the finding that phosphorylation of PRAS40 at Thr-246 is mediated by Akt in response to insulin (Kovacina et al., 2003; Nascimento et al., 2010). The presence of Akt independent phosphorylation of PRAS40 has also been reported (Lv et al., 2017; Sanchez Canedo et al., 2010). The finding that MK-2206 treatment blocked the cardioprotective effect of HDL and completely abolished Akt phosphorylation in HDL-treated WKY and SHR indicates the requirement of Akt in HDL-mediated cardiac protection. In addition, the reduction in PRAS40 phosphorylation in the presence of Akt antagonist indicates that PRAS40 is a downstream target of Akt. The presence of residual 5.7% phosphorylated PRAS40 in the presence of MK-2206 suggests the presence of, a minor, Akt-independent phosphorylation of PRAS40 in SHR (Figure 5D).

Interestingly, our data indicate that HDL activates Akt2 but not Akt1 in WKY and SHR (Figures 5B,C). Moreover, the magnitude of HDL-induced activation of Akt2 was consistent with the magnitude of HDL-mediated cardiac protection against I/R injury in WKY and SHR. HDL was more potent in activating Akt2 in WKY and resulted in greater protection from I/R injury in these rats. HDL treatment, however, did not affect PRAS40 phosphorylation in hearts from normotensive rats. The finding that HDL specifically activated Akt2 isoform yet did not induce PRAS40 phosphorylation, could possibly suggest that PRAS40 phosphorylation is likely to be mediated by Akt1 or Akt3 isoforms in WKY. In line with this observation, the lack of Akt2 did not affect the phosphorylation state of PRAS40 (Lv et al., 2017). Moreover, slicing Akt3 but not Akt1 or Akt2 blocked PRAS40 phosphorylation (Sun et al., 2020), indicating the involvement of Akt3 in PPRAS40 phosphorylation. In addition, the role of Akt3 in mediating PRAS40 phosphorylation was reported in malignant melanoma (Madhunapantula et al., 2007). A lack of HDL-induced activation of Akt1 (our data), or possibly Akt3 (remains to be tested), may therefore explain the lack of HDL-induced PRAS40 phosphorylation in WKY. The finding that HDL activated Akt2 isoform and reduced the phosphorylation of PRAS40 in hearts from SHR further supports the notion that Akt2 isoform does not play a significant role in the phosphorylation of PRAS40. HDL-mediated reduction in PRAS40 phosphorylation in SHR could alternatively be due to HDL-induced activation of phospho-protein phosphatases. Perturbation of plasma membrane cholesterol has been shown to regulate the activity of PP2A/HePTP phosphatase complex (Wang et al., 2003). PRAS40 activity is regulated by phospho-protein phosphatases including PTEN and MAPK-phosphatase-7 (MKP7) (Du et al., 2014; Wang et al., 2020). Thus, it's plausible to speculate that HDL-mediated cholesterol efflux (Rosenson et al., 2012) may enhance the activity of these phosphatases resulting in reduced PRAS40 phosphorylation. These possibilities, however, remain to be directly tested. To conclude, Akt plays a non-dispensable role in mediating the phosphorylation of PRAS40 in WKY and SHR. HDL appears to differentially regulate PRAS40 in WKY and SHR. In WKY HDL did not affect PRAS40 phosphorylation, in SHR however, HDL attenuated PRAS40 phosphorylation. HDL-mediated reduction in PRAS40 phosphorylation in SHR indicates the enhanced association of un-phosphorylated PRAS40 (active inhibitor) with mTORC1 and subsequent complex inhibition which could possibly be required to suppress the enhanced mTORC1 activity in SHR (Figures 3C, 5D). It has been reported that mTORC1 phosphorylates PRAS40 at Ser-183, Ser-212, and Ser-221 and alleviates PRAS40 induced substrate competition (Wang et al., 2008). The effect of HDL on PRAS40 phosphorylation on other sites remains however to be investigated. The finding that HDL specifically activated Akt2 suggests a novel role of Akt2 in HDL-mediated cardiac protection in normotensive and hypertensive rats. In contrast however, HDL mediated activation of Akt1 and Akt2 has been implicated in HDL-mediated protection against doxorubicin induced apoptosis (Durham et al., 2018). The lack of involvement of Akt1 in HDL-mediated protection against I/R injury could be due to species (WKY and SHR vs C57BL6 mice), model (*ex-vivo* vs *in vitro*), or pathway (I/R injury vs doxorubicin induced apoptosis) related differences. Apoptosis can be initiated through the extrinsic pathway that involves caspase 8, initiator caspase, (Tummers and Green, 2017), or via the intrinsic mitochondrial pathway, which involves mPTP opening, cytochrome c release, and caspase 7, executioner caspase, activation (Lakhani et al., 2006; Riedl and Salvesen, 2007). Akt phosphorylates and inactivates mediators of cellular apoptosis including inhibits mPTP opening, cytochrome c release, and activation of caspases (Tsang et al., 2004). HDL inactivated GSK and reduced cytochrome c release, caspases 7 and 8 activation (Figure 6).

Our data are consistent with the previously reported data on the anti-apoptotic effects of HDL (Frias et al., 2013; White et al., 2016). In addition to its anti-apoptotic effects, HDL could protect against I/R injury by virtue of its antioxidant (Calabresi et al., 2003; Fogelman et al., 2013; Mineo et al., 2006) and anti-inflammatory (Al-Jarallah and Babiker, 2022; Barter et al., 2004; Gomaraschi et al., 2008) effects. The cardioprotective anti-inflammatory, and antioxidant effects of HDL were not investigated in this study, nonetheless, they cannot be excluded.

To our knowledge, this is the first study to demonstrate the role of HDL in regulating mTORC1 and mTORC2 signaling in protecting against myocardial I/R injury in normotensive and hypertensive rats. HDL inhibited mTORC1 in normotensive and hypertensive rats yet, via different mechanisms. HDL activated mTORC2, indicated by increased Akt2 phosphorylation in WKY and SHR. HDL-mediated inhibition of mTORC1, activation of mTORC2, and inhibition of myocardial apoptosis could explain HDL-mediated cardiac protection from I/R injury in normotensive and hypertensive rats.

Our study, however, has some limitations including the rat’s age, gender, and dosage of HDL treatment used. Additional studies in female rats are required to demonstrate if HDL is equally protective in both genders. It also will be interesting to test if HDL can protect hearts from older rats, with marked hypertension-induced deterioration in cardiac functions from I/R injury or if different concentrations and/or routes of HDL administration protect to different extents.

## Data Availability

The original contributions presented in the study are included in the article/Supplementary Material, further inquiries can be directed to the corresponding author.

## Supplementary material

SUPPLEMENTARY FIGURE 1 Hearts were isolated and divided into four groups (n=4-9 rat/group from each genotype). Group-A (untreated controls) was subjected to 30 min of ischemia followed by 30 min of reperfusion. Group-B (antagonist) was infused 5 min before reperfusion and continued for an additional 10 min of reperfusion. In group-C (HDL) was subjected to 30 min of ischemia in which HDL was added 5 min before the beginning of reperfusion. HDL administration was continued during the first 10 min of reperfusion. In Group-D (antagonist + HDL) hearts were subjected to ischemia in the presence of MK-2206 (5 µM) infused at 20 min of ischemia followed by the addition of HDL (400 µg) 5 min before reperfusion. HDL and antagonist administration continued during the first 10 min of reperfusion.

## References

[B1] Abeyrathna P. Su Y. (2015). The critical role of Akt in cardiovascular function. Vasc. Pharmacol. 74, 38–48. PubMed PMID: 26025205; PubMed Central PMCID: PMCPMC4659756. 10.1016/j.vph.2015.05.008 PMC4659756 26025205

[B2] Akhtar N. Jabeen I. (2018). Pharmacoinformatic approaches to design novel inhibitors of protein kinase B pathways in cancer. Curr. Cancer Drug Targets 18 (9), 830–846. 10.2174/1568009617666170623104540 28669343

[B3] Al-Jarallah A. Babiker F. (2022). High density lipoprotein reduces blood pressure and protects spontaneously hypertensive rats against myocardial ischemia-reperfusion injury in an SR-BI dependent manner. Front. Cardiovasc Med. 9, 825310. PubMed PMID: 35387446; PubMed Central PMCID: PMCPMC8977778. 10.3389/fcvm.2022.825310 35387446 PMC8977778

[B4] Al-Jarallah A. Babiker F. A. (2024). High-density lipoprotein signaling via sphingosine-1-phosphate receptors safeguards spontaneously hypertensive rats against myocardial ischemia/reperfusion injury. Pharmaceutics 16 (4), 497. PubMed PMID: 38675158; PubMed Central PMCID: PMCPMC11054943. 10.3390/pharmaceutics16040497 38675158 PMC11054943

[B5] Babiker F. Al-Jarallah A. Al-Awadi M. (2019). Effects of cardiac hypertrophy, diabetes, aging, and pregnancy on the cardioprotective effects of postconditioning in male and female rats. Cardiol. Res. Pract. 2019, 3403959. PubMed PMID: 31198607; PubMed Central PMCID: PMCPMC6526533. 10.1155/2019/3403959 31198607 PMC6526533

[B6] Barter P. J. Nicholls S. Rye K. A. Anantharamaiah G. M. Navab M. Fogelman A. M. (2004). Antiinflammatory properties of HDL. Circ. Res. 95 (8), 764–772. PubMed PMID: 15486323. 10.1161/01.RES.0000146094.59640.13 15486323

[B7] Benavides-Serrato A. Lee J. Holmes B. Landon K. A. Bashir T. Jung M. E. (2017). Specific blockade of Rictor-mTOR association inhibits mTORC2 activity and is cytotoxic in glioblastoma. PLoS One 12 (4), e0176599. PubMed PMID: 28453552; PubMed Central PMCID: PMCPMC5409528. 10.1371/journal.pone.0176599 28453552 PMC5409528

[B8] Calabresi L. Rossoni G. Gomaraschi M. Sisto F. Berti F. Franceschini G. (2003). High-density lipoproteins protect isolated rat hearts from ischemia-reperfusion injury by reducing cardiac tumor necrosis factor-alpha content and enhancing prostaglandin release. Circ. Res. 92 (3), 330–337. PubMed PMID: 12595346. 10.1161/01.res.0000054201.60308.1a 12595346

[B9] Chen X. Cui D. Bi Y. Shu J. Xiong X. Zhao Y. (2018). AKT inhibitor MK-2206 sensitizes breast cancer cells to MLN4924, a first-in-class NEDD8-activating enzyme (NAE) inhibitor. Cell Cycle 17 (16), 2069–2079. PubMed PMID: 30198810; PubMed Central PMCID: PMCPMC6224269. 10.1080/15384101.2018.1515550 30198810 PMC6224269

[B10] Cho H. Mu J. Kim J. K. Thorvaldsen J. L. Chu Q. Crenshaw E. B. 3rd (2001). Insulin resistance and a diabetes mellitus-like syndrome in mice lacking the protein kinase Akt2 (PKB beta). Science 292 (5522), 1728–1731. PubMed PMID: 11387480. 10.1126/science.292.5522.1728 11387480

[B11] Choo A. Y. Yoon S. O. Kim S. G. Roux P. P. Blenis J. (2008). Rapamycin differentially inhibits S6Ks and 4E-BP1 to mediate cell-type-specific repression of mRNA translation. Proc. Natl. Acad. Sci. U. S. A. 105 (45), 17414–17419. PubMed PMID: 18955708; PubMed Central PMCID: PMCPMC2582304. 10.1073/pnas.0809136105 18955708 PMC2582304

[B12] Copp J. Manning G. Hunter T. (2009). TORC-specific phosphorylation of mammalian target of rapamycin (mTOR): phospho-Ser2481 is a marker for intact mTOR signaling complex 2. Cancer Res. 69 (5), 1821–1827. PubMed PMID: 19244117; PubMed Central PMCID: PMCPMC2652681. 10.1158/0008-5472.CAN-08-3014 19244117 PMC2652681

[B13] Das A. Salloum F. N. Filippone S. M. Durrant D. E. Rokosh G. Bolli R. (2015). Inhibition of mammalian target of rapamycin protects against reperfusion injury in diabetic heart through STAT3 signaling. Basic Res. Cardiol. 110 (3), 31. PubMed PMID: 25911189; PubMed Central PMCID: PMCPMC8554777. 10.1007/s00395-015-0486-5 25911189 PMC8554777

[B14] DeBosch B. Sambandam N. Weinheimer C. Courtois M. Muslin A. J. (2006b). Akt2 regulates cardiac metabolism and cardiomyocyte survival. J. Biol. Chem. 281 (43), 32841–32851. PubMed PMID: 16950770; PubMed Central PMCID: PMCPMC2724003. 10.1074/jbc.M513087200 16950770 PMC2724003

[B15] DeBosch B. Treskov I. Lupu T. S. Weinheimer C. Kovacs A. Courtois M. (2006a). Akt1 is required for physiological cardiac growth. Circulation 113 (17), 2097–2104. PubMed PMID: 16636172. 10.1161/CIRCULATIONAHA.105.595231 16636172

[B16] Dodd M. S. Ball D. R. Schroeder M. A. Le Page L. M. Atherton H. J. Heather L. C. (2012). *In vivo* alterations in cardiac metabolism and function in the spontaneously hypertensive rat heart. Cardiovasc Res. 95 (1), 69–76. PubMed PMID: 22593200; PubMed Central PMCID: PMCPMC4617603. 10.1093/cvr/cvs164 22593200 PMC4617603

[B17] Du W. W. Yang W. Fang L. Xuan J. Li H. Khorshidi A. (2014). miR-17 extends mouse lifespan by inhibiting senescence signaling mediated by MKP7. Cell Death Dis. 5, e1355. PubMed PMID: 25077541; PubMed Central PMCID: PMCPMC4123096. 10.1038/cddis.2014.305 25077541 PMC4123096

[B18] Durham K. K. Chathely K. M. Trigatti B. L. (2018). High-density lipoprotein protects cardiomyocytes against necrosis induced by oxygen and glucose deprivation through SR-B1, PI3K, and AKT1 and 2. Biochem. J. 475 (7), 1253–1265. PubMed PMID: 29523748; PubMed Central PMCID: PMCPMC5887020. 10.1042/BCJ20170703 29523748 PMC5887020

[B19] Fernandez-Hernando C. Jozsef L. Jenkins D. Di Lorenzo A. Sessa W. C. (2009). Absence of Akt1 reduces vascular smooth muscle cell migration and survival and induces features of plaque vulnerability and cardiac dysfunction during atherosclerosis. Arterioscler. Thromb. Vasc. Biol. 29 (12), 2033–2040. PubMed PMID: 19762778; PubMed Central PMCID: PMCPMC2796372. 10.1161/ATVBAHA.109.196394 19762778 PMC2796372

[B20] Filippone S. M. Samidurai A. Roh S. K. Cain C. K. He J. Salloum F. N. (2017). Reperfusion therapy with rapamycin attenuates myocardial infarction through activation of AKT and ERK. Oxid. Med. Cell Longev. 2017, 4619720. PubMed PMID: 28373901; PubMed Central PMCID: PMCPMC5360974 conflict of interests. 10.1155/2017/4619720 28373901 PMC5360974

[B21] Fogelman A. M. Reddy S. T. Navab M. (2013). Protection against ischemia/reperfusion injury by high-density lipoprotein and its components. Circ. Res. 113 (12), 1281–1282. PubMed PMID: 24311615. 10.1161/CIRCRESAHA.113.302943 24311615

[B22] Frias M. A. Pedretti S. Hacking D. Somers S. Lacerda L. Opie L. H. (2013). HDL protects against ischemia reperfusion injury by preserving mitochondrial integrity. Atherosclerosis 228 (1), 110–116. PubMed PMID: 23497785. 10.1016/j.atherosclerosis.2013.02.003 23497785

[B23] Fu W. Hall M. N. (2020). Regulation of mTORC2 signaling. Genes (Basel) 11 (9), 1045. PubMed PMID: 32899613; PubMed Central PMCID: PMCPMC7564249. 10.3390/genes11091045 32899613 PMC7564249

[B24] Gallo G. Volpe M. Savoia C. (2021). Endothelial dysfunction in hypertension: current concepts and clinical implications. Front. Med. (Lausanne) 8, 798958. PubMed PMID: 35127755; PubMed Central PMCID: PMCPMC8811286. 10.3389/fmed.2021.798958 35127755 PMC8811286

[B25] Gomaraschi M. Calabresi L. Franceschini G. (2016). Protective effects of HDL against ischemia/reperfusion injury. Front. Pharmacol. 7, 2. PubMed PMID: 26834639; PubMed Central PMCID: PMCPMC4725188. 10.3389/fphar.2016.00002 26834639 PMC4725188

[B26] Gomaraschi M. Calabresi L. Rossoni G. Iametti S. Franceschini G. Stonik J. A. (2008). Anti-inflammatory and cardioprotective activities of synthetic high-density lipoprotein containing apolipoprotein A-I mimetic peptides. J. Pharmacol. Exp. Ther. 324 (2), 776–783. PubMed PMID: 18042829. 10.1124/jpet.107.129411 18042829

[B27] Guenzle J. Akasaka H. Joechle K. Reichardt W. Venkatasamy A. Hoeppner J. (2020). Pharmacological inhibition of mTORC2 reduces migration and metastasis in melanoma. Int. J. Mol. Sci. 22 (1), 30. PubMed PMID: 33375117; PubMed Central PMCID: PMCPMC7792954. 10.3390/ijms22010030 33375117 PMC7792954

[B28] Guo X. Yu M. Kang X. Yin H. (2011). mTOR complex 2 activation by reconstituted high-density lipoprotein prevents senescence in circulating angiogenic cells. Arterioscler. Thromb. Vasc. Biol. 31 (6), 1421–1429. PubMed PMID: 21415389. 10.1161/ATVBAHA.111.224089 21415389

[B29] Harrington L. S. Findlay G. M. Gray A. Tolkacheva T. Wigfield S. Rebholz H. (2004). The TSC1-2 tumor suppressor controls insulin-PI3K signaling via regulation of IRS proteins. J. Cell Biol. 166 (2), 213–223. PubMed PMID: 15249583; PubMed Central PMCID: PMCPMC2172316. 10.1083/jcb.200403069 15249583 PMC2172316

[B30] Huang X. Zuo L. Lv Y. Chen C. Yang Y. Xin H. (2016). Asiatic acid attenuates myocardial ischemia/reperfusion injury via akt/GSK-3β/HIF-1α signaling in rat H9c2 cardiomyocytes. Molecules 21 (9), 1248. 10.3390/molecules21091248 27657024 PMC6273770

[B31] Jacinto E. Facchinetti V. Liu D. Soto N. Wei S. Jung S. Y. (2006). SIN1/MIP1 maintains rictor-mTOR complex integrity and regulates Akt phosphorylation and substrate specificity. Cell 127 (1), 125–137. PubMed PMID: 16962653. 10.1016/j.cell.2006.08.033 16962653

[B32] Jama H. A. Muralitharan R. R. Xu C. O'Donnell J. A. Bertagnolli M. Broughton B. R. S. (2022). Rodent models of hypertension. Br. J. Pharmacol. 179 (5), 918–937. PubMed PMID: 34363610. 10.1111/bph.15650 34363610

[B33] Johnson S. C. Rabinovitch P. S. Kaeberlein M. (2013). mTOR is a key modulator of ageing and age-related disease. Nature 493 (7432), 338–345. PubMed PMID: 23325216; PubMed Central PMCID: PMCPMC3687363. 10.1038/nature11861 23325216 PMC3687363

[B34] Juggi J. S. Hoteit L. J. Babiker F. A. Joseph S. Mustafa A. S. (2011). Protective role of normothermic, hyperthermic and estrogen preconditioning and pretreatment on tumour necrosis factor-alpha-induced damage. Exp. Clin. Cardiol. 16 (2), e5–e10. Epub 2011/07/13. PubMed PMID: 21747660; PubMed Central PMCID: PMCPMC3126688. 21747660 PMC3126688

[B35] Kazi A. A. Pruznak A. M. Frost R. A. Lang C. H. (2011). Sepsis-induced alterations in protein-protein interactions within mTOR complex 1 and the modulating effect of leucine on muscle protein synthesis. Shock 35 (2), 117–125. PubMed PMID: 20577146; PubMed Central PMCID: PMCPMC2995824. 10.1097/SHK.0b013e3181ecb57c 20577146 PMC2995824

[B36] Khan M. A. Hashim M. J. Mustafa H. Baniyas M. Y. Al Suwaidi S. AlKatheeri R. (2020). Global epidemiology of ischemic heart disease: results from the global burden of disease study. Cureus 12 (7), e9349. PubMed PMID: 32742886; PubMed Central PMCID: PMCPMC7384703. 10.7759/cureus.9349 32742886 PMC7384703

[B37] Kim E. Goraksha-Hicks P. Li L. Neufeld T. P. Guan K. L. (2008). Regulation of TORC1 by Rag GTPases in nutrient response. Nat. Cell Biol. 10 (8), 935–945. PubMed PMID: 18604198; PubMed Central PMCID: PMCPMC2711503. 10.1038/ncb1753 18604198 PMC2711503

[B38] Kovacina K. S. Park G. Y. Bae S. S. Guzzetta A. W. Schaefer E. Birnbaum M. J. (2003). Identification of a proline-rich Akt substrate as a 14-3-3 binding partner. J. Biol. Chem. 278 (12), 10189–10194. PubMed PMID: 12524439. 10.1074/jbc.M210837200 12524439

[B39] Kumar C. C. Madison V. (2005). AKT crystal structure and AKT-specific inhibitors. Oncogene 24 (50), 7493–7501. PubMed PMID: 16288296. 10.1038/sj.onc.1209087 16288296

[B40] Kumar V. Wollner C. Kurth T. Bukowy J. D. Cowley A. W. Jr (2017). Inhibition of mammalian target of rapamycin complex 1 attenuates salt-induced hypertension and kidney injury in Dahl salt-sensitive rats. Hypertension 70 (4), 813–821. PubMed PMID: 28827472; PubMed Central PMCID: PMCPMC5599353. 10.1161/HYPERTENSIONAHA.117.09456 28827472 PMC5599353

[B41] Lakhani S. A. Masud A. Kuida K. Porter G. A. Jr. Booth C. J. Mehal W. Z. (2006). Caspases 3 and 7: key mediators of mitochondrial events of apoptosis. Science 311 (5762), 847–851. PubMed PMID: 16469926; PubMed Central PMCID: PMCPMC3738210. 10.1126/science.1115035 16469926 PMC3738210

[B42] Laplante M. Sabatini D. M. (2012). mTOR signaling in growth control and disease. Cell 149 (2), 274–293. PubMed PMID: 22500797; PubMed Central PMCID: PMCPMC3331679. 10.1016/j.cell.2012.03.017 22500797 PMC3331679

[B43] Li J. J. Chen J. L. (2005). Inflammation may be a bridge connecting hypertension and atherosclerosis. Med. Hypotheses 64 (5), 925–929. PubMed PMID: 15780486. 10.1016/j.mehy.2004.10.016 15780486

[B44] Liu M. Huang X. Tian Y. Yan X. Wang F. Chen J. (2020). Phosphorylated GSK‑3β protects stress‑induced apoptosis of myoblasts via the PI3K/Akt signaling pathway. Mol. Med. Rep. 22 (1), 317–327. PubMed PMID: 32377749; PubMed Central PMCID: PMCPMC7248528. 10.3892/mmr.2020.11105 32377749 PMC7248528

[B45] Loewith R. Jacinto E. Wullschleger S. Lorberg A. Crespo J. L. Bonenfant D. (2002). Two TOR complexes, only one of which is rapamycin sensitive, have distinct roles in cell growth control. Mol. Cell 10 (3), 457–468. PubMed PMID: 12408816. 10.1016/s1097-2765(02)00636-6 12408816

[B46] Lv D. Guo L. Zhang T. Huang L. (2017). PRAS40 signaling in tumor. Oncotarget 8 (40), 69076–69085. PubMed PMID: 28978182; PubMed Central PMCID: PMCPMC5620322. 10.18632/oncotarget.17299 28978182 PMC5620322

[B47] Madhunapantula S. V. Sharma A. Robertson G. P. (2007). PRAS40 deregulates apoptosis in malignant melanoma. Cancer Res. 67 (8), 3626–3636. PubMed PMID: 17440074. 10.1158/0008-5472.CAN-06-4234 17440074

[B48] Matsui T. Rosenzweig A. (2005). Convergent signal transduction pathways controlling cardiomyocyte survival and function: the role of PI 3-kinase and Akt. J. Mol. Cell Cardiol. 38 (1), 63–71. PubMed PMID: 15623422. 10.1016/j.yjmcc.2004.11.005 15623422

[B49] Mineo C. Deguchi H. Griffin J. H. Shaul P. W. (2006). Endothelial and antithrombotic actions of HDL. Circ. Res. 98 (11), 1352–1364. PubMed PMID: 16763172. 10.1161/01.RES.0000225982.01988.93 16763172

[B50] Murphy E. Steenbergen C. (2005). Inhibition of GSK-3beta as a target for cardioprotection: the importance of timing, location, duration and degree of inhibition. Expert Opin. Ther. Targets 9 (3), 447–456. PubMed PMID: 15948666. 10.1517/14728222.9.3.447 15948666

[B51] Muslin A. J. (2011). Akt2: a critical regulator of cardiomyocyte survival and metabolism. Pediatr. Cardiol. 32 (3), 317–322. PubMed PMID: 21279637. 10.1007/s00246-010-9879-2 21279637

[B52] Nagao M. Toh R. Irino Y. Nakajima H. Oshita T. Tsuda S. (2017). High-density lipoprotein protects cardiomyocytes from oxidative stress via the PI3K/mTOR signaling pathway. FEBS Open Bio 7 (9), 1402–1409. PubMed PMID: 28904868; PubMed Central PMCID: PMCPMC5586351. 10.1002/2211-5463.12279 PMC5586351 28904868

[B53] Nascimento E. B. Ouwens D. M. (2009). PRAS40: target or modulator of mTORC1 signalling and insulin action? Arch. Physiol. Biochem. 115 (4), 163–175. PubMed PMID: 19480563. 10.1080/13813450902988580 19480563

[B54] Nascimento E. B. Snel M. Guigas B. van der Zon G. C. Kriek J. Maassen J. A. (2010). Phosphorylation of PRAS40 on Thr246 by PKB/AKT facilitates efficient phosphorylation of Ser183 by mTORC1. Cell Signal 22 (6), 961–967. PubMed PMID: 20138985. 10.1016/j.cellsig.2010.02.002 20138985

[B55] Oei G. T. Huhn R. Heinen A. Hollmann M. W. Schlack W. S. Preckel B. (2012). Helium-induced cardioprotection of healthy and hypertensive rat myocardium *in vivo* . Eur. J. Pharmacol. 684 (1-3), 125–131. PubMed PMID: 22497999. 10.1016/j.ejphar.2012.03.045 22497999

[B56] Oh W. J. Jacinto E. (2011). mTOR complex 2 signaling and functions. Cell Cycle 10 (14), 2305–2316. PubMed PMID: 21670596; PubMed Central PMCID: PMCPMC3322468. 10.4161/cc.10.14.16586 21670596 PMC3322468

[B57] Oshiro N. Takahashi R. Yoshino K. Tanimura K. Nakashima A. Eguchi S. (2007). The proline-rich Akt substrate of 40 kDa (PRAS40) is a physiological substrate of mammalian target of rapamycin complex 1. J. Biol. Chem. 282 (28), 20329–20339. PubMed PMID: 17517883; PubMed Central PMCID: PMCPMC3199301. 10.1074/jbc.M702636200 17517883 PMC3199301

[B58] Pedretti S. Brulhart-Meynet M. C. Montecucco F. Lecour S. James R. W. Frias M. A. (2019). HDL protects against myocardial ischemia reperfusion injury via miR-34b and miR-337 expression which requires STAT3. PLoS One 14 (6), e0218432. PubMed PMID: 31220137; PubMed Central PMCID: PMCPMC6586303. 10.1371/journal.pone.0218432 31220137 PMC6586303

[B59] Penna C. Tullio F. Moro F. Folino A. Merlino A. Pagliaro P. (2010). Effects of a protocol of ischemic postconditioning and/or captopril in hearts of normotensive and hypertensive rats. Basic Res. Cardiol. 105 (2), 181–192. PubMed PMID: 20012872. 10.1007/s00395-009-0075-6 20012872

[B60] Pullen N. Thomas G. (1997). The modular phosphorylation and activation of p70s6k. FEBS Lett. 410 (1), 78–82. PubMed PMID: 9247127. 10.1016/s0014-5793(97)00323-2 9247127

[B61] Qin X. Jiang B. Zhang Y. (2016). 4E-BP1, a multifactor regulated multifunctional protein. Cell Cycle 15 (6), 781–786. PubMed PMID: 26901143; PubMed Central PMCID: PMCPMC4845917. 10.1080/15384101.2016.1151581 26901143 PMC4845917

[B62] Riedl S. J. Salvesen G. S. (2007). The apoptosome: signalling platform of cell death. Nat. Rev. Mol. Cell Biol. 8 (5), 405–413. PubMed PMID: 17377525. 10.1038/nrm2153 17377525

[B63] Rosenson R. S. Brewer H. B. Jr. Davidson W. S. Fayad Z. A. Fuster V. Goldstein J. (2012). Cholesterol efflux and atheroprotection: advancing the concept of reverse cholesterol transport. Circulation 125 (15), 1905–1919. PubMed PMID: 22508840; PubMed Central PMCID: PMCPMC4159082. 10.1161/CIRCULATIONAHA.111.066589 22508840 PMC4159082

[B64] Ruilope L. M. Schmieder R. E. (2008). Left ventricular hypertrophy and clinical outcomes in hypertensive patients. Am. J. Hypertens. 21 (5), 500–508. PubMed PMID: 18437140. 10.1038/ajh.2008.16 18437140

[B65] Samidurai A. Ockaili R. Cain C. Roh S. K. Filippone S. M. Kraskauskas D. (2020). Differential regulation of mTOR complexes with miR-302a attenuates myocardial reperfusion injury in diabetes. iScience 23 (12), 101863. PubMed PMID: 33319180; PubMed Central PMCID: PMCPMC7725936. 10.1016/j.isci.2020.101863 33319180 PMC7725936

[B66] Sancak Y. Thoreen C. C. Peterson T. R. Lindquist R. A. Kang S. A. Spooner E. (2007). PRAS40 is an insulin-regulated inhibitor of the mTORC1 protein kinase. Mol. Cell 25 (6), 903–915. PubMed PMID: 17386266. 10.1016/j.molcel.2007.03.003 17386266

[B67] Sanchez Canedo C. Demeulder B. Ginion A. Bayascas J. R. Balligand J. L. Alessi D. R. (2010). Activation of the cardiac mTOR/p70(S6K) pathway by leucine requires PDK1 and correlates with PRAS40 phosphorylation. Am. J. Physiol. Endocrinol. Metab. 298 (4), E761–E769. PubMed PMID: 20051528. 10.1152/ajpendo.00421.2009 20051528

[B68] Sarbassov D. D. Guertin D. A. Ali S. M. Sabatini D. M. (2005). Phosphorylation and regulation of Akt/PKB by the rictor-mTOR complex. Science 307 (5712), 1098–1101. PubMed PMID: 15718470. 10.1126/science.1106148 15718470

[B69] Sciarretta S. Volpe M. Sadoshima J. (2014). Mammalian target of rapamycin signaling in cardiac physiology and disease. Circ. Res. 114 (3), 549–564. PubMed PMID: 24481845; PubMed Central PMCID: PMCPMC3995130. 10.1161/CIRCRESAHA.114.302022 24481845 PMC3995130

[B70] Sun D. Luo T. Dong P. Zhang N. Chen J. Zhang S. (2020). M2-polarized tumor-associated macrophages promote epithelial-mesenchymal transition via activation of the AKT3/PRAS40 signaling pathway in intrahepatic cholangiocarcinoma. J. Cell Biochem. 121 (4), 2828–2838. PubMed PMID: 31692069. 10.1002/jcb.29514 31692069

[B71] Tsang A. Hausenloy D. J. Mocanu M. M. Yellon D. M. (2004). Postconditioning: a form of “modified reperfusion” protects the myocardium by activating the phosphatidylinositol 3-kinase-Akt pathway. Circ. Res. 95 (3), 230–232. PubMed PMID: 15242972. 10.1161/01.RES.0000138303.76488.fe 15242972

[B72] Tummers B. Green D. R. (2017). Caspase-8: regulating life and death. Immunol. Rev. 277 (1), 76–89. PubMed PMID: 28462525; PubMed Central PMCID: PMCPMC5417704. 10.1111/imr.12541 28462525 PMC5417704

[B73] Wagner C. Ebner B. Tillack D. Strasser R. H. Weinbrenner C. (2013). Cardioprotection by ischemic postconditioning is abrogated in hypertrophied myocardium of spontaneously hypertensive rats. J. Cardiovasc Pharmacol. 61 (1), 35–41. PubMed PMID: 23052031. 10.1097/FJC.0b013e3182760c4d 23052031

[B74] Wang L. Harris T. E. Lawrence J. C. Jr (2008). Regulation of proline-rich Akt substrate of 40 kDa (PRAS40) function by mammalian target of rapamycin complex 1 (mTORC1)-mediated phosphorylation. J. Biol. Chem. 283 (23), 15619–15627. PubMed PMID: 18372248; PubMed Central PMCID: PMCPMC2414301. 10.1074/jbc.M800723200 18372248 PMC2414301

[B75] Wang L. Harris T. E. Roth R. A. Lawrence J. C. Jr (2007). PRAS40 regulates mTORC1 kinase activity by functioning as a direct inhibitor of substrate binding. J. Biol. Chem. 282 (27), 20036–20044. PubMed PMID: 17510057. 10.1074/jbc.M702376200 17510057

[B76] Wang P. Y. Liu P. Weng J. Sontag E. Anderson R. G. (2003). A cholesterol-regulated PP2A/HePTP complex with dual specificity ERK1/2 phosphatase activity. EMBO J. 22 (11), 2658–2667. PubMed PMID: 12773382; PubMed Central PMCID: PMCPMC156752. 10.1093/emboj/cdg255 12773382 PMC156752

[B77] Wang W. Wang X. Guo H. Cai Y. Zhang Y. Li H. (2020). PTEN inhibitor VO-OHpic suppresses TSC2(-) (/) (-) MEFs proliferation by excessively inhibiting autophagy via the PTEN/PRAS40 pathway. Exp. Ther. Med. 19 (6), 3565–3570. PubMed PMID: 32346419; PubMed Central PMCID: PMCPMC7185083. 10.3892/etm.2020.8629 32346419 PMC7185083

[B78] White C. R. Giordano S. Anantharamaiah G. M. (2016). High-density lipoprotein, mitochondrial dysfunction and cell survival mechanisms. Chem. Phys. Lipids 199, 161–169. PubMed PMID: 27150975; PubMed Central PMCID: PMCPMC4972637. 10.1016/j.chemphyslip.2016.04.007 27150975 PMC4972637

[B79] Wullschleger S. Loewith R. Hall M. N. (2006). TOR signaling in growth and metabolism. Cell 124 (3), 471–484. PubMed PMID: 16469695. 10.1016/j.cell.2006.01.016 16469695

[B80] Yano T. Ferlito M. Aponte A. Kuno A. Miura T. Murphy E. (2014). Pivotal role of mTORC2 and involvement of ribosomal protein S6 in cardioprotective signaling. Circ. Res. 114 (8), 1268–1280. PubMed PMID: 24557881; PubMed Central PMCID: PMCPMC4006628. 10.1161/CIRCRESAHA.114.303562 24557881 PMC4006628

[B81] Yano T. Miki T. Tanno M. Kuno A. Itoh T. Takada A. (2011). Hypertensive hypertrophied myocardium is vulnerable to infarction and refractory to erythropoietin-induced protection. Hypertension 57 (1), 110–115. PubMed PMID: 21060000. 10.1161/hypertensionaha.110.158469 21060000

[B82] Yildiz M. Oktay A. A. Stewart M. H. Milani R. V. Ventura H. O. Lavie C. J. (2020). Left ventricular hypertrophy and hypertension. Prog. Cardiovasc Dis. 63 (1), 10–21. PubMed PMID: 31759953. 10.1016/j.pcad.2019.11.009 31759953

[B83] Yu H. Littlewood T. Bennett M. (2015). Akt isoforms in vascular disease. Vasc. Pharmacol. 71, 57–64. PubMed PMID: 25929188; PubMed Central PMCID: PMCPMC4728195. 10.1016/j.vph.2015.03.003 PMC4728195 25929188

[B84] Zhou X. Frohlich E. D. (2007). Analogy of cardiac and renal complications in essential hypertension and aged SHR or L-NAME/SHR. Med. Chem. 3 (1), 61–65. PubMed PMID: 17266625. 10.2174/157340607779317634 17266625

