# Supplementary material for: High-density lipoprotein protects normotensive and hypertensive rats against ischemia-reperfusion injury through differential regulation of mTORC1 and mTORC2 signaling
Source: Front Pharmacol. 2024 Nov 14;15:1398630. doi: 10.3389/fphar.2024.1398630 (PMC11603114; doi:10.3389/fphar.2024.1398630)
Supplement: Supplementary file 1 [file Image1.pdf]

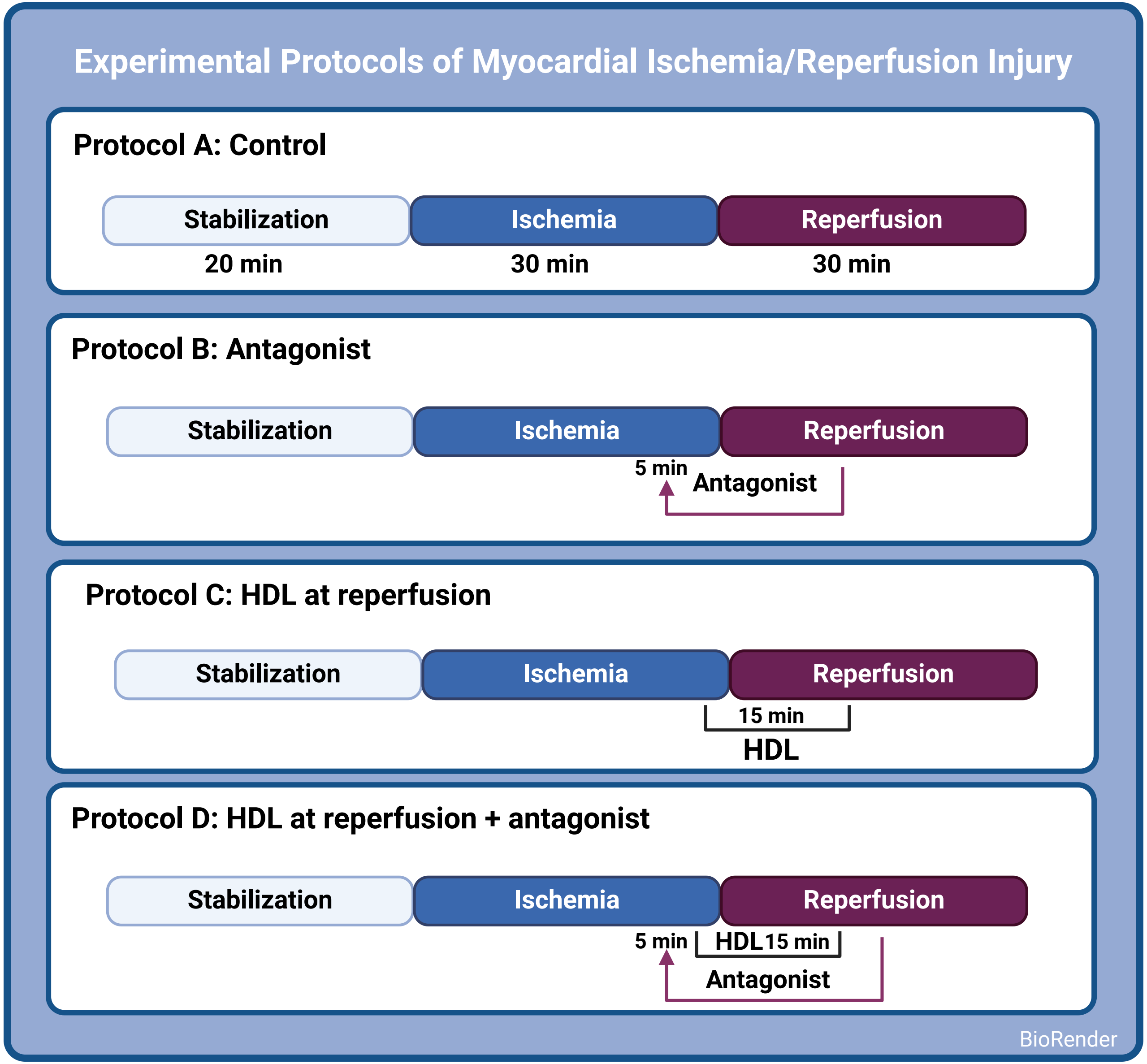
Experimental Protocols of Myocardial Ischemia/Reperfusion Injury
Protocol A: Control
Stabilization
Ischemia
Reperfusion
20 min
30 min
30 min
Protocol B: Antagonist
Stabilization
Ischemia
Reperfusion
5 min
Antagonist
Protocol C: HDL at reperfusion
Stabilization
Ischemia
Reperfusion
15 min
HDL
Protocol D: HDL at reperfusion + antagonist
Stabilization
Ischemia
Reperfusion
5 min
HDL
15 min
Antagonist
BioRender
